# Coming up short: Generative network models fail to accurately capture long-range connectivity

**DOI:** 10.1162/NETN.a.35

**Published:** 2025-11-20

**Authors:** Stuart Oldham, Alex Fornito, Gareth Ball

**Affiliations:** Developmental Imaging, Murdoch Children’s Research Institute, The Royal Children’s Hospital, Melbourne, Australia; The Turner Institute for Brain and Mental Health, School of Psychological Sciences and Monash Biomedical Imaging, Monash University, Clayton, Australia; Department of Paediatrics, University of Melbourne, Melbourne, Australia

**Keywords:** Generative network model, Long-range connections, Topography, Topology, Hubs, Brain network

## Abstract

Generative network models (GNMs) have been proposed to identify the mechanisms/constraints that shape the organization of the connectome. These models parameterize the formation of interregional connections using a trade-off between connection cost and topological complexity or biophysical similarity. Despite their simplicity, GNMs can generate synthetic networks that capture many topological properties of empirical brain networks. However, current models often fail to capture the topography (i.e., spatial embedding) of many such properties, such as the anatomical location of network hubs. In this study, we investigate a diverse array of GNM formulations and find that none can accurately capture empirical patterns of long-range connectivity. We demonstrate that the spatial embedding of longer-range connections is critical in defining hub locations and that it is precisely these connections that are poorly captured by extant models. We further show how standard measures used for model optimization and evaluation mask these and other differences between synthetic and empirical brain networks, highlighting the need for care when interpreting GNMs and metrics. Overall, our findings demonstrate common failure modes of GNMs, identify why these models do not fully capture brain network organization, and suggest ways the field can move forward to address these challenges.

## Author Summary

Generative network models aim to explain the organization of connectomes using simple wiring rules. While these models replicate topological features of brain networks, they do not capture key topographical properties, such as the anatomical location of network hubs. We show that this failure occurs because the models are unable to accurately capture the spatial position of long-range interregional connections. Moreover, standard evaluation measures fail to accurately quantify the similarity between model and empirical networks. This study identifies how and why limitations of current generative models occur and suggests ways forward for improved practices.

## INTRODUCTION

Dynamic neuronal activity coordinated across distributed brain networks supports a diverse range of complex cognitive processes. This coordination is enabled by the connectome, which comprises the complete array of neuronal connections within the brain of any organism (Bullmore & Sporns, 2009). In humans, connectomes are typically studied using noninvasive diffusion magnetic resonance imaging, which has been used to reveal a core set of complex topological properties, such as a heterogeneous distribution of connectivity across nodes (Oldham & Fornito, 2019; van den Heuvel & Sporns, 2013), small-world organization (Bassett & Bullmore, 2006, 2017), hierarchical modular architecture (Bassett et al., 2008; Meunier et al., 2009; Sporns & Betzel, 2016), and a rich-club organization in which high-degree hub nodes are strongly interconnected with each other (Arnatkevičiūtė et al., 2021; Oldham & Fornito, 2019; van den Heuvel & Sporns, 2011, 2013).

These topological properties, despite imbuing the network with a certain degree of complexity, may nonetheless arise from simple wiring principles (Albert & Barabási, 2002; Barabási & Albert, 1999; Watts & Strogatz, 1998). These principles can be studied using generative network models (GNMs), which formalize specific wiring rules that are used to construct synthetic networks (Betzel et al., 2016; Betzel & Bassett, 2017; Oldham, Fulcher, et al., 2022; Vértes, 2023; Vértes et al., 2012). Rules that give rise to networks with properties that resemble those observed in empirical data give an indication as to how connectome architecture is shaped. Therefore, generative network modelling provides a useful framework for identifying putative mechanistic or organizational principles that may underlie the formation and development of human brain networks (Betzel & Bassett, 2017; Vértes, 2023). GNMs have also been used to explore how variations in wiring rules relate to individual differences in cognitive performance and psychopathological symptoms (Akarca et al., 2021; Carozza et al., 2023; Vértes et al., 2012; Zhang et al., 2021).

Simple models that attempt to minimize the physical wiring cost of the network (often operationalized as the total connection length of the network) capture many aspects of human connectome organization (Cherniak et al., 2004; Chklovskii, 2004; Chklovskii et al., 2002; Ercsey-Ravasz et al., 2013; Henderson & Robinson, 2013, 2014; Horvát et al., 2016; Kaiser & Hilgetag, 2004; Raichle & Mintun, 2006; Rivera-Alba et al., 2011, 2014; Song et al., 2014) but often fail to capture properties associated with high-cost, long-range connections (Bullmore & Sporns, 2012; B. L. Chen et al., 2006; Kaiser & Hilgetag, 2006) that act as crucial foundations for dynamic brain function (Betzel & Bassett, 2018; Bullmore & Sporns, 2012; Deco et al., 2021; Kaiser & Hilgetag, 2006; Sato, 2021).

An alternative class of models that attempts to overcome the limitations of cost-only models are based on cost-value trade-offs (Bullmore & Sporns, 2012; Y. Chen et al., 2013; Schröter et al., 2017; van den Heuvel et al., 2016). These models often specify some trade-off between a penalty on long-range connections (i.e., to minimize wiring costs) and a bias towards forming connections that enhance topological complexity in some way (Akarca et al., 2021; Betzel et al., 2016; Carozza et al., 2023; Vértes et al., 2012; for caveats, see Oldham, Fulcher, et al., 2022). Such trade-off models typically perform better than those based on cost minimization alone, particularly when the topological bias favors a form of topological homophily, in which connections are favored between nodes with similar neighborhoods (Akarca et al., 2021; Arnatkevičiūtė et al., 2021; Betzel et al., 2016; Y. Chen et al., 2017; Goulas, Betzel, et al., 2019; Vértes et al., 2012; Zhang et al., 2021). Formulations incorporating wiring rules based on the molecular similarity of brain regions have also proven successful (Arnatkevičiūtė et al., 2021; Oldham, Fulcher, et al., 2022), following evidence that cortical regions with similar cytoarchitectural, molecular, and laminar organization are more likely to be connected (Barbas, 1986; Fornito et al., 2019; Hilgetag et al., 2019).

The biological insight offered by GNMs rests on their ability to accurately model empirical human brain networks. While adept at generating networks with similar topologies, GNMs generally fail to capture the way in which these properties are spatially embedded—that is, the topography of the connectome (Akarca et al., 2021; Arnatkevičiūtė et al., 2021; Y. Chen et al., 2017; Oldham, Fulcher, et al., 2022; Zhang et al., 2021). For instance, the models can often capture the degree distribution of the empirical data, including the existence of hubs, but the hubs reside in very different anatomical locations to those of actual connectomes (Arnatkevičiūtė et al., 2021; Oldham, Fulcher, et al., 2022). This oversight is critical given that the brain’s geometry constrains its function (Pang et al., 2023), and cortical hubs are positioned to achieve a near-optimal trade-off between wiring costs minimization and promoting complex, efficient brain dynamics (Gollo et al., 2018; Roberts et al., 2016). Topographical properties of the brain thus represent important features that generative models of brain networks should capture.

In this study, we perform a comprehensive evaluation of the ability of GNMs to reproduce both the topology and topography of human brain networks. We first demonstrate how and why GNMs fail to capture topographical features of brain networks. Secondly, we examine why the objective functions commonly used to fit GNMs are not sensitive to these differences, finding they can obscure the true extent of differences between networks and are highly sensitive to the presence of certain network properties, but not others. Our findings highlight where GNMs have weaknesses and how the choice of measures used to evaluate them may mask discrepancies with empirical connectomes. By identifying these weaknesses, we outline potential future avenues to address them.

## RESULTS

### Generative Models of the Human Connectome

**Figure 1.**
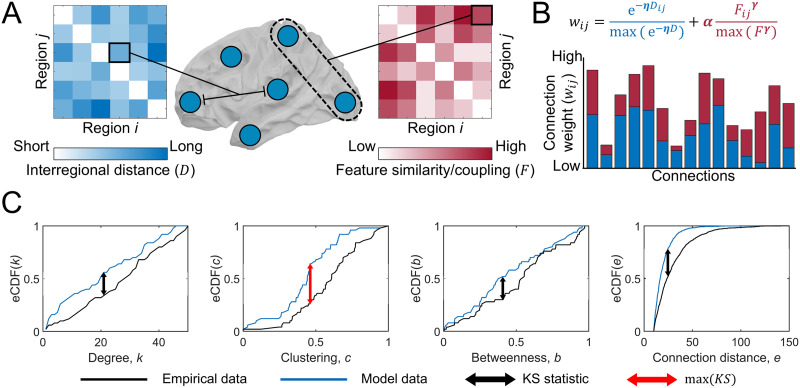
Schematic of the generative network model and max(*KS*) calculation. (A) The distance (wiring cost; *D*_*ij*_) and similarity/coupling on a given feature (*F*_*ij*_) between nodes *i* and *j* is used in the generative network model. *F*_*ij*_ can represent either interregional similarity on some biophysiological feature or coupling of some topological feature. (B) Each connection is assigned a weight *w*_*ij*_ according to the equation shown, each defines a trade-off between the cost of a potential connection (e^−*ηD*_*ij*_^) against the regional similarity *F*_*ij*_^*γ *^. Each term is normalised by its respective maximum value. Three parameters *η*, *γ*, and *α* control the strength of the distance penalty, the scaling of the feature pairwise values, and the relative contribution of the feature term respectively. The model adds connections on an iterative basis to a network based on *w*_*ij*_ (higher values indicate a greater likelihood of forming, bar plot). (C) Topological similarity between empirical and synthetic networks is defined as the maximum KS statistic max(*KS*) across four topological distributions: degree, clustering, betweenness, and connection distance. The arrows indicate where the KS statistic is calculated; the red arrow indicates the maximal difference across the four properties (i.e., max(*KS*)).

We defined GNMs using a standard formulation (Betzel et al., 2016; Oldham, Fulcher, et al., 2022). Given a network of *n* nodes, the models add connections sequentially to populate an empty *n* × *n* adjacency matrix, *A*, according to the wiring rule

$$w_{ij}=\frac{{\text{e}}^{-\eta D_{ij}}}{\max\left({\text{e}}^{-\eta D}\right)}+\alpha \frac{{F_{ij}}^{\gamma }}{\max\left(F^{\gamma }\right)},$$ (1)

where *D*_*ij*_ is the wiring distance between nodes *i* and *j*, and *F*_*ij*_ is a pairwise interaction for a given feature (either biophysiological similarity or topological coupling). The parameters *η*, *γ*, and *α* control the strength of the distance penalty, the scaling of the feature pairwise values, and the relative contribution of the feature term, respectively. Each term is normalized by the maximum value of connections that are not already present in the network. The value of *w*_*ij*_ is then used to inform the probability of a given connection being generated at each timestep of the model (Figures 1A and 1B; Methods; alternative formulations were explored in the Supporting Information).

The parameters of the model were formulated to always impose a penalty of long-range connections (the strength of which could vary), while affording the flexibility to form connections between regions with similar or dissimilar nodal properties (Oldham, Fulcher, et al., 2022). The value of *F*_*ij*_ could indicate either a measure of pairwise similarity for a cortical feature (e.g., correlation of two regions gene expression patterns), or indicate some level of topological coupling (e.g., the matching index, which quantifies the similarity of the topological neighborhoods between nodes) between the pair of regions that is continuously updated as the model progressively adds connections to the network.

We defined a total of 10 different GNMs (Table 1), which included: one where pairwise similarities between nodes *F*_*ij*_ was defined using the matching index (*Matching*); seven with similarity defined by different cortical biophysiological features, which include similarity in gene expression, receptor density, lamination, glucose uptake, haemodynamic activity, electrophysiological activity, and temporal profiles (Hansen et al., 2023); and two baseline models, one using wiring distance only (*Spatial*); and one where nodal similarity was based on random, spatially autocorrelated data (*Random similarity*). We focused on these 10 models as: (a) previous work has found that biophysiological similarity GNMs outperform topological GNMs (Arnatkevičiūtė et al., 2021; Oldham, Fulcher, et al., 2022); (b) these seven biophysiological measures represent a comprehensive summary of different measurements of interregional similarity (Hansen et al., 2023); (c) the *Matching* model is often found to be the best performing topological model so was included as a point of comparison; and (d) the two baseline models are appropriate nulls given the impact of distance and spatial autocorrelations in shaping cortical features and connectivity. In the Supporting Information, we examined 12 topological GNMs (Supporting Information Table S1) that have been widely used previously (Akarca et al., 2021; Arnatkevičiūtė et al., 2021; Betzel et al., 2016; Oldham, Fulcher, et al., 2022).

**Table 1.** Description of different generative network models

GNM	Description of wiring rule
*Spatial*	Spatial distance between regions (i.e., wiring cost)
*Gene co-expression*	Interregional similarity in gene expression combined with a wiring cost
*Receptor similarity*	Interregional similarity in receptor density combined with a wiring cost
*Laminar similarity*	Interregional similarity in cortical layer histology combined with a wiring cost
*Metabolic coupling*	Interregional similarity in PET timeseries combined with a wiring cost
*Haemodynamic coupling*	Interregional similarity in fMRI timeseries combined with a wiring cost
*Electrophysiological coupling*	Interregional similarity in MEG timeseries combined with a wiring cost
*Temporal similarity*	Interregional similarity in fMRI timeseries features combined with a wiring cost
*Random similarity*	Interregional similarity in randomly generated spatially autocorrelated features combined with a wiring cost
*Matching*	Interregional similarity in topological neighborhoods combined with a wiring cost

Synthetic networks generated by each GNM were compared to a consensus 200 region left hemisphere network, constructed using diffusion tractography from 326 unrelated participants of the Human Connectome Project (HCP) (Hansen et al., 2023). Topological similarity between model and empirical connectomes was assessed via the max(*KS*) statistic—which is the maximum Kolmogorov–Smirnov (KS) statistic (Dudley, 2015) between the node degree, node betweenness, node clustering, and connection length cumulative distribution functions for a pair of networks (Figure 1C; see Methods). Model parameters were fitted by optimizing the *max*(*KS*) statistic. Topographical similarity was measured by connection recovery (*R*), defined as the proportion of empirical connections that the model network successfully recaptured, and the Spearman correlation (*ρ*) between model and empirical network’s nodal degree (Methods).

### GNMs Capture Topological But Not Topographical Properties of Empirical Networks

**Figure 2.**
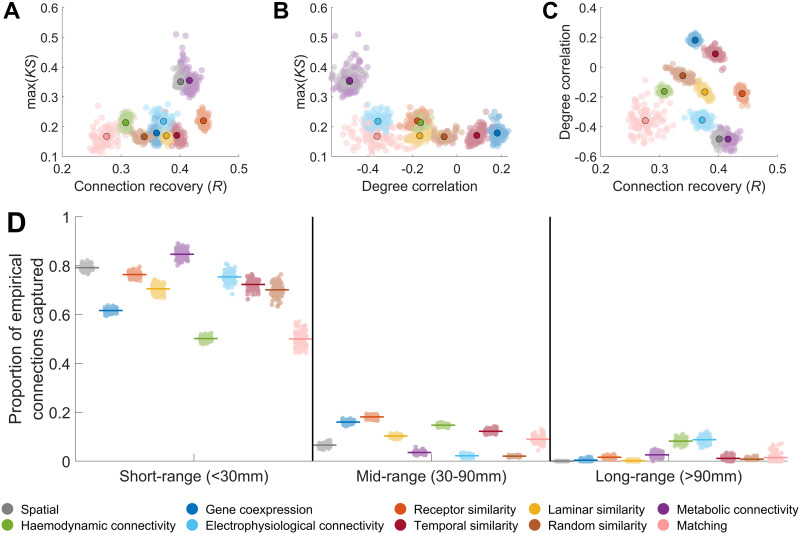
Performance of generative network models in capturing topological and topographical properties. (A) Relationship between max(*KS*) and connection recovery of synthetic model networks. (B) Relationship between max(*KS*) and the correlation between empirical and model node degree. (C) Relationship between connection recovery and the correlation between empirical and model degree. In each plot, the outlined point indicates the average across model runs for different model formulations. (D) Proportion of empirical short-range, mid-range, and long-range connections captured by the best fitting generative network models. The colored line indicates the average overlap, while each point indicates the result for an individual model network.

The best fit to empirical network topology, indicated by lowest mean max(*KS*), was achieved by the *Random similarity* (*M* = 0.166, *SD* = 0.012), *Matching* (*M* = 0.168, *SD* = 0.030), and *Laminar similarity* (*M* = 0.169, *SD* = 0.013) GNMs (Figures 2A and 2B). The *Matching* GNM could produce networks with the lowest max(*KS*) values (0.11), but not consistently. All models achieved mean max(*KS*) < 0.4, with values closer to 0 indicating a greater topological fit (Figure 2).

The best max(*KS*) value we observed was in line with previous observations using GNMs (Akarca et al., 2021; Arnatkevičiūtė et al., 2021; Betzel et al., 2016; Oldham, Fulcher, et al., 2022). Despite this comparable performance in capturing network topology, the mean connection overlap between empirical and synthetic networks ranged between only 28% and 44% across all GNM formulations (Figure 2A). Similar performance was observed across alternative GNM formulations (Supporting Information Figures S1–S2). Degree topography was also poorly captured, with Spearman correlations between empirical and model degree sequences ranging from −0.48 to 0.18 (Figure 2B–C; Supporting Information Figures S1–S2).

We observed a strong distance dependence in connection recovery. Short-range empirical connections (<30 mm) were well captured (50%–85%), but recovery dropped to 2%–18% for mid-range (30–90 mm) and 0%–9% for long-range (>90 mm) connections (Figure 2B; Supporting Information Figures S3–S4). Examining the false discovery rate of connections formed by the GNMs (i.e., the proportion of generated connections that were not in the empirical data), further indicated that the majority (>80%) of all connections formed at mid- and long-distance scales were not observed empirically (Supporting Information Figure S5). These findings suggest that GNMs can produce synthetic networks with similar topological properties to empirical connections but fail to accurately model long-range connections, leading to poor topographical correspondence between model and data. We also examined the properties of networks produced by the GNMs that had the highest degree sequence correlations. As expected, these networks had a slightly better degree topography (*ρ* ≈ 0.25), but connection recovery remained low (≈20%; Supporting Information Figure S6), and long-range connections were still poorly captured (Supporting Information Figure S7).

**Figure 3.**
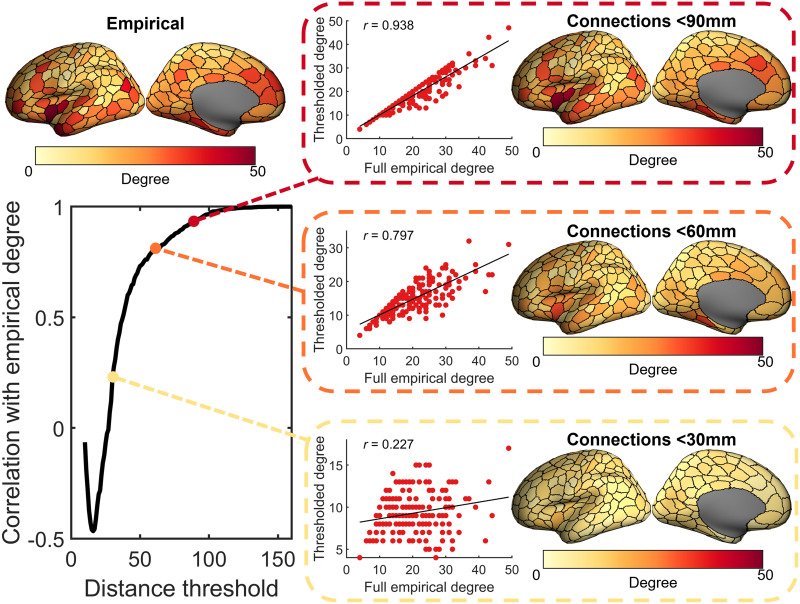
Similarity of the empirical degree sequence at different distance thresholds. Degree is calculated only using connections with a length less than the current distance threshold. The thresholded degree is then correlated (Pearson correlation) with the full empirical degree (i.e., degree calculated when using all empirical connections). Degree is largely shaped by connections >30 mm (particularly mid-range connections), meaning that these specific connections need to be captured to obtain the empirical spatial embedding of nodal degree.

Long-range connections are essential for defining nodal degree topography. To demonstrate this, we applied a progressive connection-length thresholding procedure to the empirical network. When retaining only short-range connections (<30 mm), the degree sequence was only weakly correlated with the unthresholded network (*r* = 0.23). As longer connections were incrementally added, correlation with the original degree sequence increased substantially (Figure 3). In contrast, removing short connections (<60 mm) had minimal impact on degree topography (Supporting Information Figure S8), indicating that long-range connectivity—especially among hub regions—plays a central role in shaping cortical degree distribution, despite representing only ≈26% of all connections.

Finally, while our primary focus was on left-hemisphere models, we also tested the performance of the 10 primary GNMs on whole-brain networks. Results were broadly consistent with the single-hemisphere findings: max(*KS*) values ranged from 0.16 to 0.59, and degree correlations from −0.48 to 0.06. However, connection recovery was lower overall (0.25 < *R* < 0.40; Supporting Information Figure S9), and these also did not show any notable improvement in capturing mid- or long-range connections, but models based on gene-expression and receptor similarity could capture ≈40% of mid-range connections (Supporting Information Figure S10).

**Figure 4.**
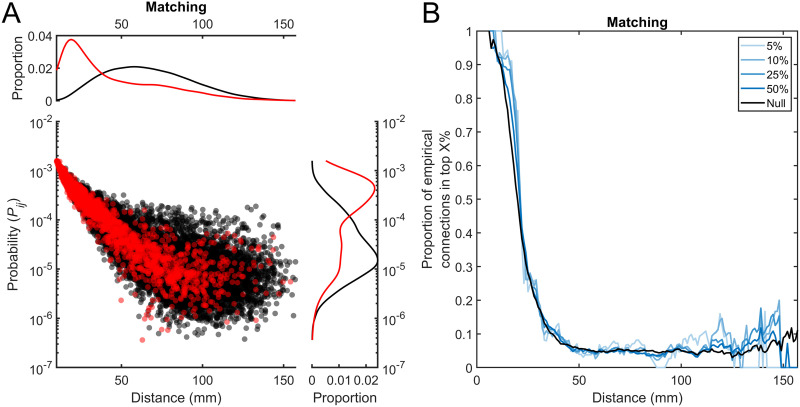
Connection probabilities of the *Matching* model. (A) Connection probabilities for the *Matching* generative network model. The connection probabilities are the mean probability assigned to each connection across all steps of the model. Red points indicate empirical connections (i.e., connections that exist in the empirical data) while black points indicate empirical nonconnections (i.e., connections that do not exist in the empirical data). Kernal density plots for the structural and nonstructural connections are shown for the distributions of distance/length and probability against the respective axis. (B) Most probable connections at different distances. Using a sliding window analysis, at each distance (±5 mm) the top X% of connections with the highest probability are found. The proportion of these that are empirical connections is then calculated. The “null” line indicates the proportion of empirical structural connections that exist at that distance (i.e., the probability of selecting an empirical structural connection if all connections at a given distance were equally probable).

To determine why GNMs do not accurately model long-range connections between hub nodes, we calculated the probability of each connection (*P*_*ij*_) during model fitting as a function of connection length. In all models, *P*_*ij*_ was negatively associated with connection length (Figure 4A; Supporting Information Figure S11). While at short distances, almost all edges assigned high probability by the model are connected in the empirical networks, at longer ranges, only a small proportion of high probability edges are connected (Figure 4B). At connection lengths over 60 mm, only ≈6% of the top 25% most probable connections exist in the empirical data across all GNMs, a similar proportion to that expected by selecting edges at random (≈5%; Figure 4B). As such, GNMs are highly unlikely to accurately identify the long-range connections between empirical hub nodes (Supporting Information Figure S12).

### The max(*KS*) Fit Statistic Can Obscure Important Differences in Network Structure

**Figure 5.**
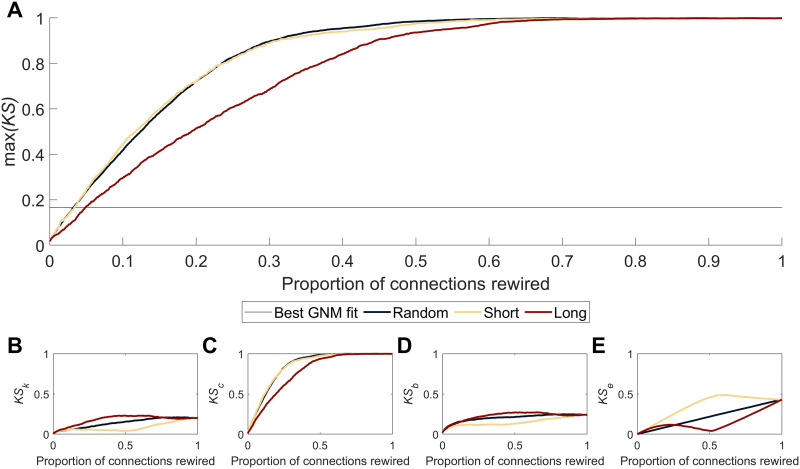
Effect of iteratively rewiring connections on max(*KS*). (A) The proportion of connections rewired and its effect of max(*KS*) for different rewiring algorithms. The *random* algorithm rewired connections at random but of a similar length; *short* rewired connections from shortest to longest connections with those of a similar length and; *long* rewired connections from longest to shortest connections with those of a similar length. The dotted line indicates the lowest max(*KS*) fit obtained in the GNMs. Plots also show the change in the *KS* statistics for the degree (B), clustering (C), betweenness (D), and connection length (E) distributions (*KS*_*k*_, *KS*_*c*_, *KS*_*b*_, and *KS*_*e*_ respectfully). Changes in max(*KS*) were almost exclusively driven by changes in clustering.

Our results have demonstrated that maximizing topological similarity between two networks through minimization of the max(*KS*) statistic can result in widely different network topographies and poor correspondence between empirical and synthetic network connections (Figure 2A–C). GNMs were unable to recreate the empirical topographic layout of degree or long-range connections, yet the topological similarity of empirical and synthetic connectomes as measured by the max(*KS*) statistic remains high. To understand this disconnect, we sought to quantify how variations in the max(*KS*) statistic relates to differences in network topology and topography. To do so, we incrementally rewired connections in the empirical human brain network—either at random or based on connection length—and measured max(*KS*) between the rewired and original networks (see Methods, Figure 5).

We observed that max(*KS*) increased rapidly as connections were rewired (Figure 5A), such that rewiring of just 64 connections resulted in a max(*KS*) of 0.166 with 97% of empirical connections retained (Figure 5A). This value is comparable to the max(*KS*) of the best-performing GNMs (Figure 2), which only replicated at most 44% of connections. Constraining the algorithm to only rewire long connections, thus leaving shorter connections in place, reduced this effect with max(*KS*) most sensitive to changes in shorter connections (Figure 5A). When we examined how the correlation between the nodal degree of the original and rewired network varied across iterations, we found that rewiring long-range connections caused a greater decrease in the correlation than rewiring short-range connections (Supporting Information Figure S13).

The max(*KS*) statistic is a composite of four different topological properties (degree, clustering, betweenness, connection length; Figure 1). Examining the KS statistic for each of these four properties, we found that the increased max(*KS*) largely reflected changes in the network clustering coefficient (Figure 5B–E). The rewiring analysis indicates max(*KS*) is highly sensitive to small perturbations in network connectivity that disrupt network clustering.

While the pattern of topological and topographical features is relatively conserved across individual empirical brain networks, there is still individual variation. To establish how this variation is reflected in variations of the max(*KS*) statistics in empirical connectomes, we compared 972 individual networks constructed from HCP data, using 10 parcellations (Schaefer 100 to 1000) and deterministic or probabilistic tractography (see Methods). All networks produced using a given parcellation and tractography algorithm were matched for the same density. We fitted the 10 GNM types to the ensemble of individual empirical networks generated with the Schaefer 400 parcellation. For each individual, we (a) identified the synthetic network with the lowest max(*KS*) value and (b) calculated max(*KS*) with respect to every other empirical network as well.

**Figure 6.**
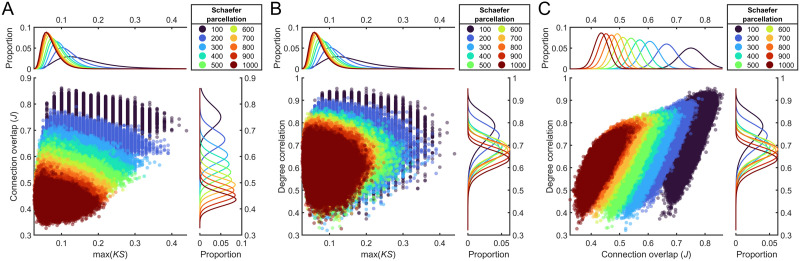
Performance of max(*KS*), connection overlap, and similarity of the degree distribution when comparing empirical networks produced using probabilistic tractography. (A) Relationship between max(*KS*) and connection overlap. (B) Relationship between max(*KS*) and the correlation between two empirical networks nodal degree. (C) Relationship between connection overlap and the correlation between two empirical networks nodal degree. The kernel density plots show the distributions for each parcellation on the respective feature.

We found that the similarity between empirical brain networks, as measured by the max(*KS*) statistic, varied depending on parcellation size, with finer parcellations showing greater consistency across individuals (Figure 6). Overall, max(*KS*) varied from 0.025 to 0.44 between individual empirical network pairs, with connection overlaps (Jaccard index) ranging from 0.33 to 0.86.

Both topological and topographical similarity varied with parcellation resolution. For example, the Schaefer 100 parcellation had a mean max(*KS*) of 0.15 (*SD* = 0.039) compared to the Schaefer 400 parcellation (0.09, *SD* = 0.028) and the Schaefer 1000 parcellation (0.070, *SD* = 0.022). Empirical networks created using coarser parcellations also showed greater connection overlap between individuals (e.g., Schaefer 100: *M* = 0.752, *SD* = 0.025; Schaefer 400: *M* = 0.571, *SD* = 0.024; Schaefer 1000: *M* = 0.435, *SD* = 0.021), in addition to higher degree correlations (e.g., Schaefer 100: *M* = 0.765, *SD* = 0.065; Schaefer 400: *M* = 0.685, *SD* = 0.052; Schaefer 1000: *M* = 0.63, *SD* = 0.043), than finer resolution parcellations (Figure 6). Similar results were obtained when using deterministic tractography (Supporting Information Figure S14).

**Figure 7.**
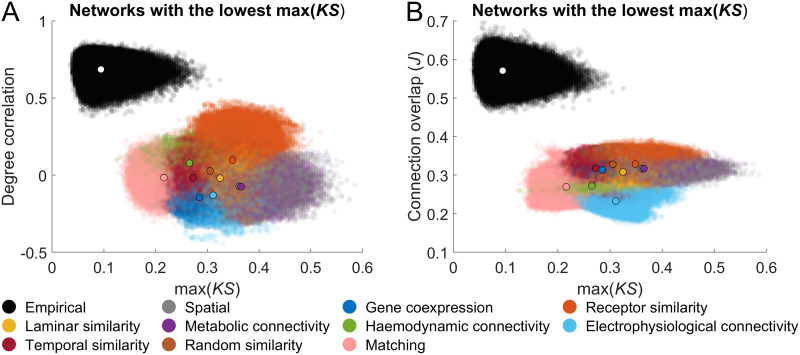
Performance of max(*KS*) in empirical networks. Scatter plots depict the relationship between max(*KS*) and the (A) degree correlation or (B) connection overlap for both empirical-synthetic (GNM-produced vs. empirical networks) and empirical-empirical (empirical network vs. empirical network) comparisons. In each plot, the black-outlined dot represents the mean value for each models empirical-synthetic comparisons, with the white dot indicating the mean for empirical-empirical comparisons. The networks with the lowest max(*KS*) value for each participant were extracted, and their similarity to all other empirical participant networks was computed. Additionally, all pairs of individual empirical networks were compared using each similarity metric. All empirical networks were generated via probabilistic tractography and using the Schaefer 400 parcellation.

On average, topological similarity (as measured by max(*KS*)) between individual empirical and synthetic networks generally occupied a similar range to interindividual similarity, with over 96% of *Matching, Gene co-expression*, and *Temporal similarity* GNM networks within the range of interindividual empirical-empirical values (other models showed 10%–89% overlap; Figure 7A). However, for measures of topography (i.e., connection overlap and degree correlation), empirical-synthetic network comparisons showed almost no overlap with the range of values recorded in empirical-empirical networks (Figure 7B). Overall, this result demonstrates that topographic similarity can show wide variation even within a constrained range of max(*KS*) values.

### Different Similarity Measures Do Not Identify More Topographically Similar Networks

Our analysis reveals that the max(*KS*) statistic is highly sensitive to small changes in short-range connectivity (Figure 5), but is not sensitive to large differences in long-range connectivity. We next assessed if alternative measures of network similarity could identify networks with greater topological and topographic similarity to empirical data (Supporting Information Tables S2–S3). These alternatives were informed by approaches proposed by other studies (Akarca et al., 2025; Goñi et al., 2013; Liu et al., 2024), including *TND* (similarity across global measures of topology), *TF*_*diff*_ (Euclidean norm of the difference between correlation matrices of topological features), max(*r*_*d*_) (maximum Pearson distance across degree/clustering/betweenness/connection length), and max(*RMSE*) (maximum mean-root-squared error across degree/clustering/closeness/connection length).

**Figure 8.**
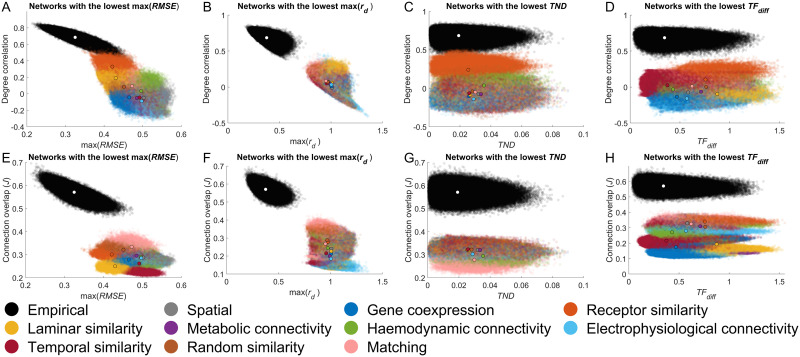
Performance of different similarity measures in generative network models (GNMs) and empirical networks. Scatter plots depict the relationship between degree correlation and (A) max(*RMSE*); (B) max(*r*_*d*_); (C) *TND*; and (D) *TF*_*diff*_ for both empirical-synthetic (GNM-produced vs. empirical networks) and empirical-empirical (empirical network vs. empirical network) comparisons. Similarly, scatter plots show the relationship between the connection overlap and (E) max(*RMSE*); (F) max(*r*_*d*_); (G) *TND*; and (H) *TF*_*diff*_ for the same comparisons. In each plot, the black-outlined dot represents the mean value for each models empirical-synthetic comparisons, with the white dot indicating the mean for empirical-empirical comparisons. For each similarity measure, the networks with the lowest value for each participant were extracted, and their similarity to all other empirical participant networks was computed. Additionally, all pairs of individual empirical networks were compared using each similarity metric. All empirical networks were generated via probabilistic tractography and using the Schaefer 400 parcellation.

We first compared how the different similarity measures performed in the rewiring analysis, finding that the alternative measures showed a rate of change equal to or lower than that of max(*KS*) (Supporting Information Figure S15). Next, we computed the similarity of each network generated during the optimization using these alternative measures. While the optimization procedure was intended to maximize max(*KS*), as this procedure densely samples the entire parameter space, it allows for exploration if other parameters can produce a network similar to the empirical data. For each individual empirical network, (a) the most similar network (under each metric) generated during the optimization was selected and (b) the similarity of this network to all other individual empirical networks on given measure was calculated. As with max(*KS*), no alternative metric identified synthetic networks with both a high degree correlation and connection overlap to empirical data (Figure 8). Some measures—max(*r*_*d*_) and max(*RMSE*)—indicated that synthetic networks were distinct from empirical networks (Figures 8A, 8B, 8E, and 8F), *TND* and *TF*_*diff*_ identified networks that were as similar to empirical networks as empirical networks were to each other (mean overlap across GNMs: *TND* = 99%; *TF*_*diff*_ = 99%; Figures 8C, 8D, 8G, and 8H). Measures that indicated the generated networks were similar to empirical data primarily assessed topological properties, whereas those that suggested dissimilarity also accounted for topographical differences. None of the alternative measures successfully identified networks with strong topographical similarity to empirical data. Together, these findings suggest that alternative objective functions are unlikely to improve the topographical correspondence between GNM-generated and empirical networks.

## DISCUSSION

GNMs offer a powerful framework for investigating potential mechanisms and constraints of human brain network organization. Nonetheless, our results establish that many popular topological GNMs routinely fail to capture critical topographical network organization due to inaccurate reconstruction of long-range connections between cortical hubs. This is because traditional measures used for model optimization and evaluation based on the similarity of network topology are biased toward the reconstruction of short-range connections. GNMs prioritizing similarity of regional anatomical, cellular, molecular, or dynamical features also fail to overcome this bias, with selection of edges based on similarity in random data showing comparable performance. Our findings therefore indicate that current approaches for defining and fitting GNMs do not identify any single model or parameter combination that can produce a network which accurately captures both the topology and topography of empirical connectome data.

Our analysis indicates that a major reason for the noted failure of extant GNMs to capture connectome topography is their inability to reproduce the specific node-node pairing of long-range connections—a failure that is particularly evident when examining the degree sequence of empirical data (Arnatkevičiūtė et al., 2021; Y. Chen et al., 2017; Oldham, Fulcher, et al., 2022; Zhang et al., 2021) is that the models do not reproduce the specific node-node pairing of long-range connections, which play a critical role in shaping the rich-club organization of the brain (Arnatkevičiūtė et al., 2021; Fulcher & Fornito, 2016; van den Heuvel et al., 2012; van den Heuvel & Sporns, 2011, 2013). This is because these connections do not have a major impact on the max(*KS*) statistic, which is the objective function most often used to optimize GNM parameters.

In the GNMs evaluated here, long-range connections impart significant wiring cost, *D*_*ij*_. A high value of interregional or topological similarity, *F*_*ij*_, is therefore required to overcome this distance penalty and increase the probability of connection. In almost every formulation tested, empirically connected and unconnected edges could not be clearly distinguished based on a given feature at longer distances (Figure 4A). A partial exception to this was long-range interhemispheric connections, who, in certain models (e.g., gene co-expression), were highly replicated, likely because homotopic regions have similar biophysiological properties that help promote long-range connections between them. However, for most other long-range connections, the extent of biological similarity between unconnected regions was similar to or greater than the similarity of connected regions. This observation appears to stand in contrast to previous studies demonstrating that structurally connected cortical areas are on average more similar to each other than to unconnected areas (Arnatkevic̆iūtė et al., 2018, 2021; Fulcher & Fornito, 2016; Hansen et al., 2023; Hilgetag et al., 2019; Wei et al., 2018), or that hubs regions share similar patterns of gene expression and cortical structure (Arnatkevičiūtė et al., 2021; Paquola et al., 2019, 2020; Sydnor et al., 2021). However, the extent of biological assortativity may vary with distance in the brain, with short range connections existing between biologically similar regions and long-range connections being between biologically dissimilar regions (Bazinet et al., 2023; Betzel & Bassett, 2018; Markov et al., 2013). Currently, GNMs cannot model such a distance dependency, which may partially account for why long-range connections cannot be accurately captured.

Compounding the issue of GNMs accurately capturing connectivity is that max(*KS*), the primary measure used to determine network similarity, is sensitive to differences in short-range, but not long-range connectivity. Short-range connections most heavily contribute to defining max(*KS*); therefore, GNMs that can accurately capture the arrangement of short-range connections will perform well on this measure. This accounts for why many models achieve a low max(*KS*) as the most probable connections are short-range, even when based on randomly spatially autocorrelated features (Figure 4C; Supporting Information Figure S8). This also means that different GNMs can produce very different networks to each other, yet be gauged as equally similar to the empirical data merely because they have accurately captured short-range connections. Conversely, if a network accurately recaptures long-range connectivity, and therefore likely topographical properties, but not short-range connectivity, it will do poorly on max(*KS*). In either case, it is clear that max(*KS*) does not provide a complete picture of the data, thus using it as an objective function on it to identify the most similar network to the modeled data may therefore result in overly optimistic estimates of the extent to which a given model captures desired empirical properties. Even if current GNMs were adapted to use an objective function sensitive to topography (e.g., max(*RMSE*)), our results suggest they would still fall short of accurately reproducing the full topography of empirical connectomes, due to fundamental limitations in the model framework itself.

Even though for the current class of GNMs an alternative objective function is unlikely to dramatically improve their performance, future work should take care when selecting an optimization function as this decision is nontrivial (Betzel & Bassett, 2017). For example, if the objective function was defined as connection overlap, this could result in a synthetic network that accurately recaptures many empirical connections but has a radically different topology (Betzel et al., 2016; Betzel & Bassett, 2017). However, our results show that the opposite is also true, two networks can have a very similar topology despite few overlapping edges. Objective functions are selected on the basis they are capturing key relevant properties of the network (Betzel et al., 2016; Betzel & Bassett, 2017). The class of GNMs used in this paper were developed to provide a parsimonious description of the connectomes organization with respect to its topology; therefore, the objective function (i.e., max(*KS*)) was designed to reflect this (Betzel et al., 2016). It is unlikely that optimizing for topology will produce a network with a prescribed topography, as a network with a given topology can have a large number of different topographical representations (Kaiser & Hilgetag, 2006). While important, topology alone cannot explain the entire organization of the connectome. The spatial embedding of connectivity dictates the function of brain networks (Gollo et al., 2018; Kaiser & Hilgetag, 2006; Pang et al., 2023; Roberts et al., 2016); for example, specific long-range connections are needed to promote brainlike dynamics (Vohryzek et al., 2025). Therefore, network topography is a crucial organizational property that GNMs should aim to capture. If GNMs are to be used to make stronger claims about the mechanisms and constraints of connectome organization, the objective function will likely need to be amended to reflect topographical similarity.

### Improving GNMs

Our findings indicate that current GNMs offer an insufficient account of both the topology and topography of human connectomes mapped with diffusion MRI. It is useful to reflect on the potential reasons for this failure as a way of improving the biological accuracy of the models.

One possible factor limiting the accuracy of these models is that they fail to account for regional differences in the timing with which different connections form, a phenomenon known as developmental heterochronicity (Beul et al., 2018; Goulas, Betzel, et al., 2019; Goulas, Majka, et al., 2019; Hilgetag et al., 2019). Differential timing of cortical network development has been suggested as a mechanism underlying the emergence of network features like hubs and long-range connections (Kaiser, 2017; Oldham, Ball, et al., 2022; Oldham & Fornito, 2019). Prior work indicates that incorporating global developmental changes in geometry can improve model fits (Oldham, Fulcher, et al., 2022), but imbuing GNMs with regionally patterned windows within which specific connections are formed may be critical for shaping the degree topography of the human brain. Indeed, it has been noted that areas with poorer laminar differentiation complete neurogenesis and start forming connections earlier than areas with more pronounced differentiation (Beul et al., 2017; Cahalane et al., 2012; Epihova et al., 2024; Finlay & Uchiyama, 2015; Scholtens et al., 2014; van den Heuvel et al., 2015). Since network hubs tend to be located in transmodal regions that show poorer differentiation than primary sensory cortices (Buckner et al., 2009; Margulies et al., 2016), they may obtain an early advantage that drives a rich-get-richer phenomenon known to play a critical role in the emergence of network hubs in a diverse array of systems (Barabási & Albert, 1999; Nicosia et al., 2013).

Furthermore, the best way to parameterize interregional variability in biophysiological features remains unclear. Although cytoarchitectonic similarity has been proposed as a key criterion for predicting if two regions are connected (Arnatkevičiūtė et al., 2021; Barbas, 2015; Beul et al., 2017, 2018; Hilgetag et al., 2019; Oldham, Fulcher, et al., 2022), some evidence suggests that dissimilar regions may be more likely to be connected across long distances (Bazinet et al., 2023; Betzel & Bassett, 2018; Markov et al., 2013); models may need to account for a distance-dependent homophilic attachment mechanism. In some GNMs based on biophysiological similarity, interhemispheric connections could be recaptured with greater accuracy than long-range intrahemispheric ones, suggesting that long-range inter- and intrahemispheric connectivity may be governed by different attachment mechanisms. Additionally, our models did not directly examine how homophilic attachment based on biophysiological properties may interact with homophilic attachment based on topological properties. Configuring the models in such a way may expand their ability to capture complex connectivity patterns.

An alternative way forward for GNMs is to move away from models focused on pairwise interactions between regions to capture dynamic properties such as axonal growth (Goulas, Betzel, et al., 2019; Liu et al., 2024; Song et al., 2014). Such models have been implemented for simple 2D or 3D representations of the cortical surface (Goulas, Betzel, et al., 2019; Liu et al., 2024; Song et al., 2014) but could be extended to incorporate the complex geometry of the human brain. These more biophysical models would also have the advantage of easily allowing for connection weights to be modeled, and potentially allow for the shape of the formed tracts to be examined (Liu et al., 2024), providing additional angles with which to evaluate the synthetically produced connectivity.

### Limitations

Several biases have been identified that can limit the biological veracity of empirical connectome data derived from diffusion MRI (Sotiropoulos & Zalesky, 2019). This includes an inherent difficulty in capturing long-range connections (Sotiropoulos & Zalesky, 2019), thus the empirical networks employed in this study may underestimate the true extent of long-range connections in human brain networks. Despite this, GNMs in their current form are still unable to reach this lower bound. Multiple approaches have been developed to mitigate some of the biases in diffusion MRI tractography and network construction (Baum et al., 2018; Oldham et al., 2020), yet variations between methods can still yield networks with major differences in topology and topography (Gajwani et al., 2023; Oldham et al., 2020). While it is possible that observations on GNMs applied to data processed using one pipeline may not generalize to data processed in other ways, we believe that the identified issues with GNMs persist across different processing choices. Prior work using GNMs for networks constructed using deterministic tractography revealed similar findings (Oldham, Fulcher, et al., 2022). We also found that, for networks created using distance-dependent thresholding, which explicitly aims to preserve long-range connections, or strength thresholding, which can decrease estimates of long-range connectivity, GNMs were unable to model the correct positioning of hubs. Furthermore, when we extended our analysis to whole-brain connectivity (as opposed to a single hemisphere), which includes substantially more long-range connections, similar weaknesses were observed. Finally, GNMs have been used to model primate structural brain networks based on tract tracing data (Y. Chen et al., 2017), with similar observations to those obtained in tractography-based human brain networks, most notably a failure to capture hub location and long-range connections.

We did not consider the weighted topology/topography of empirical connectomes, which is a salient feature of brain networks (Fornito et al., 2016; Markov et al., 2011). One recent GNM has been proposed to model connection weights, and shown promise in capturing binary and weighted features (Akarca et al., 2023), although it remains to be seen if the reported model fits, which rely on a similar approach to that investigated here, are subject to the same biases. Our models were restricted to just cortical connections, and thus did not account for the complex connectivity patterns linking the cortex, subcortex, and cerebellum (Müller et al., 2020; Oldham et al., 2025; Oldham & Ball, 2023; Park et al., 2024; Raut et al., 2020; Tian et al., 2020). Expanding GNMs to include all types of brain regions is needed to achieve a more comprehensive understanding of mechanisms underpinning all connectome organization.

### Conclusion

GNMs can successfully replicate basic topological properties of brain networks but they fall short in capturing topographical properties, particularly the crucial long-range connections observed in empirical data. Additionally, the common practice of evaluating model fits purely based on topology may overlook the true extent of differences between networks. Our findings establish where and why GNMs struggle in capturing brain connectivity and indicate directions for future research to address these challenges and improve model accuracy.

## METHODS

### Empirical Brain Networks

Two empirical datasets were used in this study, a group averaged connectivity matrix (Hansen et al., 2023) and a set of individualized connectivity matrices (Arnatkevičiūtė et al., 2021; Oldham, Fulcher, et al., 2022). Both datasets were constructed from HCP data (Glasser et al., 2013). The group averaged data had been constructed from the S900 release of the HCP data from 326 participants. In brief, the preprocessed diffusion data had been processed with MRtrix3 using multishell, multitissue constrained spherical deconvolution and tractography was conducted with second-order Integration over Fiber Orientation Distributions (iFOD2). Streamlines were weighted using SIFT2 (Smith et al., 2015), and the group consensus connectivity matrix was created using a distance-dependent thresholding algorithm. Further details are available in Hansen et al. (2023). The group connectivity matrix was binarized for use in this study. The dataset was originally created for the full Schaefer 400, 7-network parcellation (Schaefer et al., 2018), which includes 200 regions for each hemisphere. We primarily used data for the left hemisphere, that is, 200 cortical regions in total as to: (a) reduce computational complexity (more nodes/connections in the network increases the time needed to run the GNM), (b) reduce any bias that may result of measuring the Euclidean distance from nodes across hemispheres, and (c) keep consistent with previous methodologies (Arnatkevičiūtė et al., 2021; Betzel et al., 2016; Oldham, Fulcher, et al., 2022). However, we also examined how a limited number of GNMs performed in recapturing connections/connectivity patterns in the original whole brain network.

The individualized connectivity data had also previously been processed (Arnatkevičiūtė et al., 2021; Oldham, Fulcher, et al., 2022). These data were constructed from the S1200 release of the HCP for 973 participants. A similar series of processing steps were applied as with the first dataset. In addition to iFOD2, a probabilistic tractography algorithm, the deterministic algorithm, Fiber Assignment by Continuous Tractography, was also run. Further details of the processing steps for the individualized data are available elsewhere (Arnatkevičiūtė et al., 2021; Oldham, Fulcher, et al., 2022). We generated a network for each of the Schaefer 7-network 100–1000 parcellations, using the probabilistic and deterministic tractograms resulting in 20 networks per participant (two different tractograms across 10 different parcellations). As with the group averaged data, we only retained regions in the left hemisphere. One of the 973 individual networks showed noticeably altered connectivity compared to others (i.e., lower density, weaker correlations with other individuals), thus they were excluded from further analysis.

Networks were either unthresholded or thresholded based on connection strength. Thresholding was applied such that only the strongest *E* connections were retained, where *E* was 70% of the minimum number of connection observed for any individual in a given tractography/parcellation combination. While a strength threshold is likely to result in weak, long-range connections being pruned (Fornito et al., 2016), we used this approach to ensure that all individuals for a given network type had the same density with some variation in their topology. Other thresholding approaches involve the creation of a consensus matrix, which is then used as a mask for individuals (Betzel et al., 2019; de Reus & van den Heuvel, 2013; Roberts et al., 2017), but such approaches would minimize interindividual variability in network binary network topology and thus meaning there would be little value in comparing individual binary network organization.

### Cortical Interregional Similarity Measures

We used seven different measures of interregional similarity which represent multiple facets of brain organization (Hansen et al., 2023): (a) gene co-expression (correlation across 8,687 genes); (b) receptor similarity (correlation across 18 PET receptor density profiles); (c) laminar similarity (correlation across cortical layer histology); (d) metabolic coupling (correlation in PET timeseries); (e) haemodynamic coupling (correlation in fMRI timeseries); (f) electrophysiological coupling (correlation in MEG timeseries); and (g) temporal similarity (correlation across 6,441 fMRI timeseries features). These measures had previously been generated for the Schaefer 400 parcellation and made openly available as part of another study (see Hansen et al., 2023, for details). All measures of interregional similarity were provided as a matrix of Pearson correlations. For use with GNMs, each correlation matrix was converted to a Pearson distance (as GNMs cannot handle negative values for interregional similarity) and then rescaled to the unit interval.

These regional measures are representative of major organizational patterns in the cortex (Hansen et al., 2023; Markello et al., 2022). However, these measures can demonstrate spatial autocorrelations, which can explain many properties of the brain (Burt et al., 2020; Markello & Misic, 2021; Shinn et al., 2023). To assess the impact of spatial autocorrelations in GNMs, we created random spatially autocorrelated features and computed interregional similarity on those using the following procedure:

1. We generated a random value in the range 0–1 for each cortical region.
2. For each cortical region, we took the average value of each of the nodes it was adjacent to on the cortical surface.
3. We then updated the value of each cortical region to this averaged value (note this was only done once all the averaged values for all regions had been calculated).
4. Steps 2–3 were repeated five times.
5. Steps 1–4 were repeated 20 times to create 20 random spatially autocorrelated features.
6. Random interregional similarity was calculated by performing a Pearson correlation across each of these random features for every pair of brain regions.

The above steps were performed for regions of the left hemisphere only. To map the spatially autocorrelated patterns to the right hemisphere (as the parcellation is not homotopic across hemispheres), the left hemisphere random features were mapped to the right hemisphere, and the mean value was taken for each right hemisphere region.

While there are more sophisticated approaches of creating spatially autocorrelated cortical features (Burt et al., 2020), this approach is a fast and efficient way of creating multiple such maps. As with the other measures of interregional similarity, the Pearson correlation was converted to a Pearson distance and then rescaled to the unit interval. In addition to these measures of interregional similarity, we also examined 12 topologically based models (i.e., topological coupling was between a pair of regions was used for the value of *F*_*ij*_; Supporting Information Table S1).

Additionally Euclidean distances between all pairs of nodes were calculated to get an estimate of wiring costs for the GNM. Specifically, the Euclidean distance was taken as the average distance from all the vertices on the cortical surface that were assigned to one region, to all the vertices assigned to another region. Euclidean distances do not account for the curved paths tracts have to take, that is, fiber distances, and so can underestimate the true wiring costs. While more elaborate procedures exist for approximating the fiber lengths and therefore wiring costs (Oldham, Fulcher, et al., 2022), Euclidean and fiber distances are often strongly correlated (Betzel et al., 2016), and using either to predict connections in brain networks has resulted in similar performance (Rubinov et al., 2015). We therefore deemed Euclidean distances as an acceptable approximation of wiring costs for within hemisphere connectivity.

### GNM Formulation

The GNM adds connections in a sequential manner up to the desired number (i.e., the number of connections in the network the model is attempting to replicate) according to the wiring rule specified in Equation 1. As indicated by this equation, GNMs were primarily formulated as an additive rule, and the distance decay was defined according to an exponential. This additive rule has previously been shown to more accurately capture the trade-offs between model parameters implied by the model than multiplicative formulations (Oldham, Fulcher, et al., 2022). Alternatively, the rule could be formulated as a multiplication of terms

$$w_{ij}=\frac{{\text{e}}^{-\eta D_{ij}}}{\max\left({\text{e}}^{-\eta D}\right)}\times \frac{{F_{ij}}^{\gamma }}{\max\left(F^{\gamma }\right)}.$$ (2)

While we primarily model distance as an exponential decay e^−*ηD*_*ij*_^ due to evidence showing the likelihood of a connection existing decreases exponential as a function of its length, other studies have used a power-law decay *D*_*ij*_^−*η*^ (Betzel et al., 2016), but the specific form of the distance penalty does not change the basic conclusions of our findings.

At each timestep of the model, the weight *w*_*ij*_ is converted to a probability according to

$$P_{ij}=\frac{w_{ij}\left(1-A_{ij}\right)}{\sum_{u}\sum_{v>u}w_{uv}\left(1-A_{uv}\right)},$$ (3)

where (1 − *A*_*ij*_) ensures that only connections that are not yet present in the network influence the connection probability (i.e., the probability of a connection forming should only be relative to other connections that have not formed yet). At each timestep, a single connection is established, with *P*_*ij*_ indicating the probability that specific connection will be selected. If *F*_*ij*_ is a measure of topology, that measure will be recalculated based on the network will the newly added connection.

As *w*_*ij*_ will change at each timestep of the model, so will *P*_*ij*_. Therefore, to calculate the overall probability a given connection will form, we average connection probabilities across model timesteps (note the average was only calculated for iterations where the connection had not been added into the network).

### Selecting Optimized GNMs

To determine the optimal values for the parameters *η*, *γ*, and *α* for each GNM, we employed a previously developed optimization method (Betzel et al., 2016; Oldham, Fulcher, et al., 2022). The method was implemented as follows:

1. We randomly sampled 2,000 points within the parameter space defined by *η*, *γ*, and potentially *α* (if the additive form was used). When an exponential was used, *η* varied between 0 and 2, while for a power-law *η* varied between 0 and 10. For GNMs using interregional similarity, *γ* varied between −20 and 200. For those based on topology, *γ* varied between −10 and 10. Additionally, *α* ranged from 0 to 10 (when α was included as a parameter in the model). These parameter ranges were selected based on initial testing and previous findings (Oldham, Fulcher, et al., 2022).
2. At each sampled point representing a specific combination of *η*, *γ*, and *α* values, we generated a network using the newly defined parameters, resulting in 2,000 synthetic networks. We calculated the max(*KS*) statistic for each generated network. The max(*KS*) statistic is the maximum *KS* value (i.e., greatest discrepancy) between the generated and empirical networks in terms of their degree, clustering, betweenness centrality, and connection length distributions.
3. After evaluating all networks, we employed Voronoi tessellation to identify regions (cells) of the parameter space associated with low fit statistics. An additional 2,000 points in the parameter space were preferentially sampled from each cell based on the relative probability $VC_{c}^{-\beta }$, where *VC*_*c*_ is the max(*KS*) of cell *c*, and *β* controls the likelihood of sampling from cells with low max(*KS*) values.
4. Steps 2 and 3 were repeated four times, resulting in a total of 10,000 points being evaluated. In each repetition, the probability of sampling cells with better fits was increased by varying *β* from 0.5 to 2 in 0.5 increments, thus converging to an approximate optimum. When the optimization was applied to the ensemble of individual networks, the network generated by the model for a given parameter combination was compared to all individual networks (Liu et al., 2023). The max(*KS*) for that parameter combination was taken as the mean of all individual max(*KS*) values. The networks generated during the optimization that produced the lowest max(*KS*) for an individual participant were used for subsequent analyses. When evaluating other similarity measures, we used the networks generated when optimising for max(*KS*) because (a) there was a greater computational cost to running the GNM again with a different fit statistics and (b) the parameter space is densely sampled so examining from the networks selected is likely to allow the identification of a minimum.

An advantage of this approach is that it allows for the entire parameter space to be adequately sampled and facilitate the identification of a global (approximate) optimum. For the group-averaged consensus network, when the optimal parameters had been identified for a given GNM, they were used to generate 100 further networks.

### Rewiring Model

Networks were rewired at random, or from the shortest-to-longest connections, or longest to shortest. Existing connections could only be rewired/swapped with a nonexisting connection. Once a connection had been rewired, it could not be rewired again. Connections could be rewired with a selected nonexisting connection at random, a connection of a similar length, or a connection of dissimilar length. The rewiring continued until all existing connections had been rewired. We ran each rewiring model 30 times and then took the mean max(*KS*) across these runs.

### Measures of Network Topology/Topography

Details of the various topological/topographical measures used in this study can be found in Supporting Information Tables S1, S2, and S3.

## ACKNOWLEDGMENTS

Data were provided by the Human Connectome Project, WU-Minn Consortium (Principal Investigators: David Van Essen and Kamil Ugurbil; 1U54MH091657) funded by the 16 NIH Institutes and Centers that support the NIH Blueprint for Neuroscience Research and by the McDonnell Center for Systems Neuroscience at Washington University. S.O. is supported by the Brain and Behavior Research Foundation (ID: 31471). A.F. was supported by the Sylvia and Charles Viertel Foundation, National Health and Medical Research Council (IDs: 1197431 and 1146292), and Australian Research Council (IDs: DP200103509). G.B. was support by the National Health and Medical Research Council (ID: 1194497). This research was supported by the Murdoch Children’s Research Institute, the Royal Children’s Hospital, Department of Paediatrics, The University of Melbourne and the Victorian Government’s Operational Infrastructure Support Program. The project was generously supported by The Royal Children’s Hospital Foundation devoted to raising funds for research at The Royal Children’s Hospital.

## SUPPORTING INFORMATION

Supporting information for this article is available at https://doi.org/10.1162/NETN.a.35.

## AUTHOR CONTRIBUTIONS

Stuart Oldham: Conceptualization; Data curation; Formal analysis; Funding acquisition; Investigation; Methodology; Visualization; Writing – original draft; Writing – review & editing. Alex Fornito: Writing – review & editing. Gareth Ball: Conceptualization; Supervision; Writing – original draft; Writing – review & editing.

## FUNDING INFORMATION

Stuart Oldham, Brain and Behavior Research Foundation (https://dx.doi.org/10.13039/100000874), Award ID: 31471. Alex Fornito, National Health and Medical Research Council (https://dx.doi.org/10.13039/501100000925), Award ID: 1197431. Alex Fornito, National Health and Medical Research Council (https://dx.doi.org/10.13039/501100000925), Award ID: 1146292. Alex Fornito, Sylvia and Charles Viertel Charitable Foundation (https://dx.doi.org/10.13039/100008717). Gareth Ball, National Health and Medical Research Council (https://dx.doi.org/10.13039/501100000925), Award ID: 1194497. Alex Fornito, Australian Research Council (https://dx.doi.org/10.13039/501100000923), Award ID: DP200103509.

## CODE AND DATA AVAILABILITY

All analysis was performed in MATLAB 2023b. Code and data are available from https://github.com/StuartJO/ComingUpShort.

## Supplementary Material



## TECHNICAL TERMS

Hub: A node with many more connections than other nodes in the network.
Generative network model (GNM): A computational model that creates networks according to a given wiring rule.
Wiring rule: An expression dictating how different features shape the likelihood of a connection forming.
Wiring cost: The material cost (e.g., metabolic, space restrictions) of forming and maintaining an anatomical connection.
Cost-value trade-off: The notion that a network form due to pressure between minimizing wiring costs and promoting valuable connections.
Topology: Structural features of the network (e.g., the presence of hubs, an exponential decay of connection lengths).
Topography: The spatial embedding of topological features (e.g., the location of hubs, long-range connections).
Biophysiological similarity: The similarity of two nodes on a biophysiological feature (e.g., gene expression, electrophysiological activity, cortical lamination).
Topological coupling: A measure of interaction between a pair of nodes based on their topological features (e.g., mean degree, maximum clustering, number of shared neighbors).
Spatial autocorrelation: The tendency of nearby brain regions to have similar values for a given feature.
max(*KS*): The maximum Kolmogorov–Smirnov (KS) statistic between the node degree, node betweenness, node clustering, and connection length cumulative distribution functions for a pair of networks.

## References

[bib1] Akarca, D., Dunn, A. W. E., Hornauer, P. J., Ronchi, S., Fiscella, M., Wang, C., … Schröter, M. (2025). Homophilic wiring principles underpin neuronal network topology in vitro. eLife, 14, e85300. 10.7554/eLife.85300, 40626695 PMC12563572

[bib2] Akarca, D., Schiavi, S., Achterberg, J., Genc, S., Jones, D. K., & Astle, D. E. (2023). A weighted generative model of the human connectome. bioRxiv. 10.1101/2023.06.23.546237

[bib3] Akarca, D., Vértes, P. E., Bullmore, E. T., CALM team, & Astle, D. E. (2021). A generative network model of neurodevelopmental diversity in structural brain organization. Nature Communications, 12(1), 4216. 10.1038/s41467-021-24430-z, 34244490 PMC8270998

[bib4] Albert, R., & Barabási, A. L. (2002). Statistical mechanics of complex networks. Reviews of Modern Physics, 74(1), 47–97. 10.1103/RevModPhys.74.47

[bib5] Arnatkevičiūtė, A., Fulcher, B. D., Oldham, S., Tiego, J., Paquola, C., Gerring, Z., … Fornito, A. (2021). Genetic influences on hub connectivity of the human connectome. Nature Communications, 12(1), 4237. 10.1038/s41467-021-24306-2, 34244483 PMC8271018

[bib6] Arnatkevičiūtė, A., Fulcher, B. D., Pocock, R., & Fornito, A. (2018). Hub connectivity, neuronal diversity, and gene expression in the Caenorhabditis elegans connectome. PLoS Computational Biology, 14(2), e1005989. 10.1371/journal.pcbi.1005989, 29432412 PMC5825174

[bib7] Barabási, A.-L., & Albert, R. (1999). Emergence of scaling in random networks. Science, 286(5439), 509–512. 10.1126/science.286.5439.509, 10521342

[bib8] Barbas, H. (1986). Pattern in the laminar origin of corticocortical connections. Journal of Comparative Neurology, 252(3), 415–422. 10.1002/cne.902520310, 3793985

[bib9] Barbas, H. (2015). General cortical and special prefrontal connections: Principles from structure to function. Annual Review of Neuroscience, 38, 269–289. 10.1146/annurev-neuro-071714-033936, 25897871

[bib10] Bassett, D. S., & Bullmore, E. T. (2006). Small-world brain networks. Neuroscientist, 12(6), 512–523. 10.1177/1073858406293182, 17079517

[bib11] Bassett, D. S., & Bullmore, E. T. (2017). Small-world brain networks revisited. Neuroscientist, 23(5), 499–516. 10.1177/1073858416667720, 27655008 PMC5603984

[bib12] Bassett, D. S., Bullmore, E., Verchinski, B. A., Mattay, V. S., Weinberger, D. R., & Meyer-Lindenberg, A. (2008). Hierarchical organization of human cortical networks in health and schizophrenia. Journal of Neuroscience, 28(37), 9239–9248. 10.1523/JNEUROSCI.1929-08.2008, 18784304 PMC2878961

[bib13] Baum, G. L., Roalf, D. R., Cook, P. A., Ciric, R., Rosen, A. F. G., Xia, C., … Satterthwaite, T. D. (2018). The impact of in-scanner head motion on structural connectivity derived from diffusion MRI. NeuroImage, 173, 275–286. 10.1016/j.neuroimage.2018.02.041, 29486323 PMC5911236

[bib14] Bazinet, V., Hansen, J. Y., Vos De Wael, R., Bernhardt, B. C., van den Heuvel, M. P., & Misic, B. (2023). Assortative mixing in micro-architecturally annotated brain connectomes. Nature Communications, 14(1), 2850. 10.1038/s41467-023-38585-4, 37202416 PMC10195875

[bib15] Betzel, R. F., Avena-Koenigsberger, A., Goñi, J., He, Y., de Reus, M. A., Griffa, A., … Sporns, O. (2016). Generative models of the human connectome. NeuroImage, 124, 1054–1064. 10.1016/j.neuroimage.2015.09.041, 26427642 PMC4655950

[bib16] Betzel, R. F., & Bassett, D. S. (2017). Generative models for network neuroscience: Prospects and promise. Journal of the Royal Society Interface, 14(136), 20170623. 10.1098/rsif.2017.0623, 29187640 PMC5721166

[bib17] Betzel, R. F., & Bassett, D. S. (2018). Specificity and robustness of long-distance connections in weighted, interareal connectomes. Proceedings of the National Academy of Sciences, 115(21), E4880–E4889. 10.1073/pnas.1720186115, 29739890 PMC6003515

[bib18] Betzel, R. F., Griffa, A., Hagmann, P., & Mišić, B. (2019). Distance-dependent consensus thresholds for generating group-representative structural brain networks. Network Neuroscience, 3(2), 475–496. 10.1162/netn_a_00075, 30984903 PMC6444521

[bib19] Beul, S. F., Barbas, H., & Hilgetag, C. C. (2017). A predictive structural model of the primate connectome. Scientific Reports, 7, 43176. 10.1038/srep43176, 28256558 PMC5335700

[bib20] Beul, S. F., Goulas, A., & Hilgetag, C. C. (2018). Comprehensive computational modelling of the development of mammalian cortical connectivity underlying an architectonic type principle. PLoS Computational Biology, 14(11), e1006550. 10.1371/journal.pcbi.1006550, 30475798 PMC6261046

[bib21] Buckner, R. L., Sepulcre, J., Talukdar, T., Krienen, F. M., Liu, H., Hedden, T., … Johnson, K. A. (2009). Cortical hubs revealed by intrinsic functional connectivity: Mapping, assessment of stability, and relation to Alzheimer’s disease. Journal of Neuroscience, 29(6), 1860–1873. 10.1523/JNEUROSCI.5062-08.2009, 19211893 PMC2750039

[bib22] Bullmore, E., & Sporns, O. (2009). Complex brain networks: Graph theoretical analysis of structural and functional systems. Nature Reviews Neuroscience, 10(3), 186–198. 10.1038/nrn2575, 19190637

[bib23] Bullmore, E., & Sporns, O. (2012). The economy of brain network organization. Nature Reviews Neuroscience, 13(5), 336–349. 10.1038/nrn3214, 22498897

[bib24] Burt, J. B., Helmer, M., Shinn, M., Anticevic, A., & Murray, J. D. (2020). Generative modeling of brain maps with spatial autocorrelation. NeuroImage, 220, 117038. 10.1016/j.neuroimage.2020.117038, 32585343

[bib25] Cahalane, D. J., Charvet, C. J., & Finlay, B. L. (2012). Systematic, balancing gradients in neuron density and number across the primate isocortex. Frontiers in Neuroanatomy, 6, 28. 10.3389/fnana.2012.00028, 22826696 PMC3399120

[bib26] Carozza, S., Akarca, D., & Astle, D. (2023). The adaptive stochasticity hypothesis: Modeling equifinality, multifinality, and adaptation to adversity. Proceedings of the National Academy of Sciences, 120(42), e2307508120. 10.1073/pnas.2307508120, 37816058 PMC10589678

[bib27] Chen, B. L., Hall, D. H., & Chklovskii, D. B. (2006). Wiring optimization can relate neuronal structure and function. Proceedings of the National Academy of Sciences, 103(12), 4723–4728. 10.1073/pnas.0506806103, 16537428 PMC1550972

[bib28] Chen, Y., Wang, S., Hilgetag, C. C., & Zhou, C. (2013). Trade-off between multiple constraints enables simultaneous formation of modules and hubs in neural systems. PLoS Computational Biology, 9(3), e1002937. 10.1371/journal.pcbi.1002937, 23505352 PMC3591279

[bib29] Chen, Y., Wang, S., Hilgetag, C. C., & Zhou, C. (2017). Features of spatial and functional segregation and integration of the primate connectome revealed by trade-off between wiring cost and efficiency. PLOS Computational Biology, 13(9), e1005776. 10.1371/journal.pcbi.1005776, 28961235 PMC5645157

[bib30] Cherniak, C., Mokhtarzada, Z., Rodriguez-Esteban, R., & Changizi, K. (2004). Global optimization of cerebral cortex layout. Proceedings of the National Academy of Sciences, 101(4), 1081–1086. 10.1073/pnas.0305212101, 14722353 PMC327154

[bib31] Chklovskii, D. B. (2004). Synaptic connectivity and neuronal morphology: Two sides of the same coin. Neuron, 43(5), 609–617. 10.1016/j.neuron.2004.08.012, 15339643

[bib32] Chklovskii, D. B., Schikorski, T., & Stevens, C. F. (2002). Wiring optimization in cortical circuits. Neuron, 34(3), 341–347. 10.1016/S0896-6273(02)00679-7, 11988166

[bib34] Deco, G., Sanz Perl, Y., Vuust, P., Tagliazucchi, E., Kennedy, H., & Kringelbach, M. L. (2021). Rare long-range cortical connections enhance human information processing. Current Biology, 31(20), 4436–4448. 10.1016/j.cub.2021.07.064, 34437842

[bib33] de Reus, M. A., & van den Heuvel, M. P. (2013). Estimating false positives and negatives in brain networks. NeuroImage, 70, 402–409. 10.1016/j.neuroimage.2012.12.066, 23296185

[bib35] Dudley, R. M. (2015). Kolmogorov–Smirnov and Mann–Whitney–Wilcoxon tests. https://math.mit.edu/~rmd/465/edf-ks.pdf.

[bib36] Epihova, G., Epihov, D. Z., Akarca, D., & Astle, D. E. (2024). Molecular mechanisms driving divergent development of the human frontal and visual cortex during prenatal development. bioRxiv. 10.1101/2024.05.15.594422

[bib37] Ercsey-Ravasz, M., Markov, N. T., Lamy, C., Van Essen, D. C., Knoblauch, K., Toroczkai, Z., … Kennedy, H. (2013). A predictive network model of cerebral cortical connectivity based on a distance rule. Neuron, 80(1), 184–197. 10.1016/j.neuron.2013.07.036, 24094111 PMC3954498

[bib38] Finlay, B. L., & Uchiyama, R. (2015). Developmental mechanisms channeling cortical evolution. Trends in Neurosciences, 38(2), 69–76. 10.1016/j.tins.2014.11.004, 25497421

[bib39] Fornito, A., Arnatkevičiūtė, A., & Fulcher, B. D. (2019). Bridging the gap between connectome and transcriptome. Trends in Cognitive Sciences, 23(1), 34–50. 10.1016/j.tics.2018.10.005, 30455082

[bib40] Fornito, A., Zalesky, A., & Bullmore, E. T. (Eds.). (2016). Fundamentals of brain network analysis. Academic Press. 10.1016/C2012-0-06036-X

[bib41] Fulcher, B. D., & Fornito, A. (2016). A transcriptional signature of hub connectivity in the mouse connectome. Proceedings of the National Academy of Sciences, 113(5), 1435–1440. 10.1073/pnas.1513302113, 26772314 PMC4747775

[bib42] Gajwani, M., Oldham, S., Pang, J. C., Arnatkevičiūtė, A., Tiego, J., Bellgrove, M. A., & Fornito, A. (2023). Can hubs of the human connectome be identified consistently with diffusion MRI? Network Neuroscience, 7(4), 1326–1350. 10.1162/netn_a_00324, 38144690 PMC10631793

[bib43] Glasser, M. F., Sotiropoulos, S. N., Wilson, J. A., Coalson, T. S., Fischl, B., Andersson, J. L., … WU-Minn HCP Consortium. (2013). The minimal preprocessing pipelines for the Human Connectome Project. NeuroImage, 80, 105–124. 10.1016/j.neuroimage.2013.04.127, 23668970 PMC3720813

[bib44] Gollo, L. L., Roberts, J. A., Cropley, V. L., Di Biase, M. A., Pantelis, C., Zalesky, A., & Breakspear, M. (2018). Fragility and volatility of structural hubs in the human connectome. Nature Neuroscience, 21(8), 1107–1116. 10.1038/s41593-018-0188-z, 30038275

[bib45] Goñi, J., Avena-Koenigsberger, A., Velez de Mendizabal, N., van den Heuvel, M. P., Betzel, R. F., & Sporns, O. (2013). Exploring the morphospace of communication efficiency in complex networks. PLoS ONE, 8(3), e58070. 10.1371/journal.pone.0058070, 23505455 PMC3591454

[bib46] Goulas, A., Betzel, R. F., & Hilgetag, C. C. (2019). Spatiotemporal ontogeny of brain wiring. Science Advances, 5(6), eaav9694. 10.1126/sciadv.aav9694, 31206020 PMC6561744

[bib47] Goulas, A., Majka, P., Rosa, M. G. P., & Hilgetag, C. C. (2019). A blueprint of mammalian cortical connectomes. PLOS Biology, 17(3), e2005346. 10.1371/journal.pbio.2005346, 30901324 PMC6456226

[bib48] Hansen, J. Y., Shafiei, G., Voigt, K., Liang, E. X., Cox, S. M. L., Leyton, M., … Misic, B. (2023). Integrating multimodal and multiscale connectivity blueprints of the human cerebral cortex in health and disease. PLOS Biology, 21(9), e3002314. 10.1371/journal.pbio.3002314, 37747886 PMC10553842

[bib49] Henderson, J. A., & Robinson, P. A. (2013). Using geometry to uncover relationships between isotropy, homogeneity, and modularity in cortical connectivity. Brain Connectivity, 3(4), 423–437. 10.1089/brain.2013.0151, 23802922

[bib50] Henderson, J. A., & Robinson, P. A. (2014). Relations between the geometry of cortical gyrification and white-matter network architecture. Brain Connectivity, 4(2), 112–130. 10.1089/brain.2013.0183, 24437717

[bib51] Hilgetag, C. C., Beul, S. F., van Albada, S. J., & Goulas, A. (2019). An architectonic type principle integrates macroscopic cortico-cortical connections with intrinsic cortical circuits of the primate brain. Network Neuroscience, 3(4), 905–923. 10.1162/netn_a_00100, 31637331 PMC6777964

[bib52] Horvát, S., Gămănuţ, R., Ercsey-Ravasz, M., Magrou, L., Gămănuţ, B., Van Essen, D. C., … Kennedy, H. (2016). Spatial embedding and wiring cost constrain the functional layout of the cortical network of rodents and primates. PLOS Biology, 14(7), e1002512. 10.1371/journal.pbio.1002512, 27441598 PMC4956175

[bib53] Kaiser, M. (2017). Mechanisms of Connectome Development. Trends in Cognitive Sciences, 21(9), 703–717. 10.1016/j.tics.2017.05.010, 28610804

[bib54] Kaiser, M., & Hilgetag, C. C. (2004). Modelling the development of cortical systems networks. Neurocomputing, 58–60, 297–302. 10.1016/j.neucom.2004.01.059

[bib55] Kaiser, M., & Hilgetag, C. C. (2006). Nonoptimal component placement, but short processing paths, due to long-distance projections in neural systems. PLoS Computational Biology, 2(7), e95. 10.1371/journal.pcbi.0020095, 16848638 PMC1513269

[bib56] Liu, Y., Seguin, C., Betzel, R. F., Han, D., Akarca, D., Di Biase, M. A., & Zalesky, A. (2024). A generative model of the connectome with dynamic axon growth. Network Neuroscience, 8(4), 1192–1211. 10.1101/2024.02.23.581824, 39735503 PMC11674315

[bib57] Liu, Y., Seguin, C., Mansour, S., Oldham, S., Betzel, R., Di Biase, M. A., & Zalesky, A. (2023). Parameter estimation for connectome generative models: Accuracy, reliability, and a fast parameter fitting method. NeuroImage, 270, 119962. 10.1016/j.neuroimage.2023.119962, 36822248

[bib58] Margulies, D. S., Ghosh, S. S., Goulas, A., Falkiewicz, M., Huntenburg, J. M., Langs, G., … Smallwood, J. (2016). Situating the default-mode network along a principal gradient of macroscale cortical organization. Proceedings of the National Academy of Sciences of the United States of America, 113(44), 12574–12579. 10.1073/pnas.1608282113, 27791099 PMC5098630

[bib59] Markello, R. D., Hansen, J. Y., Liu, Z.-Q., Bazinet, V., Shafiei, G., Suárez, L. E., … Misic, B. (2022). Neuromaps: Structural and functional interpretation of brain maps. Nature Methods, 19(11), 1472–1479. 10.1038/s41592-022-01625-w, 36203018 PMC9636018

[bib60] Markello, R. D., & Misic, B. (2021). Comparing spatial null models for brain maps. NeuroImage, 236, 118052. 10.1016/j.neuroimage.2021.118052, 33857618

[bib61] Markov, N. T., Ercsey-Ravasz, M., Lamy, C., Ribeiro Gomes, A. R., Magrou, L., Misery, P., … Kennedy, H. (2013). The role of long-range connections on the specificity of the macaque interareal cortical network. Proceedings of the National Academy of Sciences, 110(13), 5187–5192. 10.1073/pnas.1218972110, 23479610 PMC3612613

[bib62] Markov, N. T., Misery, P., Falchier, A., Lamy, C., Vezoli, J., Quilodran, R., … Knoblauch, K. (2011). Weight consistency specifies regularities of macaque cortical networks. Cerebral Cortex, 21(6), 1254–1272. 10.1093/cercor/bhq201, 21045004 PMC3097985

[bib63] Meunier, D., Lambiotte, R., Fornito, A., Ersche, K. D., & Bullmore, E. T. (2009). Hierarchical modularity in human brain functional networks. Frontiers in Neuroinformatics, 3, 37. 10.3389/neuro.11.037.2009, 19949480 PMC2784301

[bib64] Müller, E. J., Munn, B., Hearne, L. J., Smith, J. B., Fulcher, B., Arnatkevičiūtė, A., … Shine, J. M. (2020). Core and matrix thalamic sub-populations relate to spatio-temporal cortical connectivity gradients. NeuroImage, 222, 117224. 10.1016/j.neuroimage.2020.117224, 32795658

[bib65] Nicosia, V., Vértes, P. E., Schafer, W. R., Latora, V., & Bullmore, E. T. (2013). Phase transition in the economically modeled growth of a cellular nervous system. Proceedings of the National Academy of Sciences, 110(19), 7880–7885. 10.1073/pnas.1300753110, 23610428 PMC3651470

[bib66] Oldham, S., Arnatkevičiūtė, A., Smith, R. E., Tiego, J., Bellgrove, M. A., & Fornito, A. (2020). The efficacy of different preprocessing steps in reducing motion-related confounds in diffusion MRI connectomics. NeuroImage, 222, 117252. 10.1016/j.neuroimage.2020.117252, 32800991

[bib67] Oldham, S., & Ball, G. (2023). A phylogenetically-conserved axis of thalamocortical connectivity in the human brain. Nature Communications, 14(1), 6032. 10.1038/s41467-023-41722-8, 37758726 PMC10533558

[bib68] Oldham, S., Ball, G., & Fornito, A. (2022). Early and late development of hub connectivity in the human brain. Current Opinion in Psychology, 44, 321–329. 10.1016/j.copsyc.2021.10.010, 34896927

[bib69] Oldham, S., & Fornito, A. (2019). The development of brain network hubs. Developmental Cognitive Neuroscience, 36, 100607. 10.1016/j.dcn.2018.12.005, 30579789 PMC6969262

[bib70] Oldham, S., Fulcher, B. D., Aquino, K., Arnatkevičiūtė, A., Paquola, C., Shishegar, R., & Fornito, A. (2022). Modeling spatial, developmental, physiological, and topological constraints on human brain connectivity. Science Advances, 8(22), eabm6127. 10.1126/sciadv.abm6127, 35658036 PMC9166341

[bib71] Oldham, S., Mansour L, S., & Ball, G. (2025). Perinatal development of structural thalamocortical connectivity. Imaging Neuroscience, 3, imag_a_00418. 10.1162/imag_a_00418, 40800876 PMC12319864

[bib72] Pang, J. C., Aquino, K. M., Oldehinkel, M., Robinson, P. A., Fulcher, B. D., Breakspear, M., & Fornito, A. (2023). Geometric constraints on human brain function. Nature, 618(7965), 566–574. 10.1038/s41586-023-06098-1, 37258669 PMC10266981

[bib73] Paquola, C., Seidlitz, J., Benkarim, O., Royer, J., Klimes, P., Bethlehem, R. A. I., … Bernhardt, B. C. (2020). A multi-scale cortical wiring space links cellular architecture and functional dynamics in the human brain. PLOS Biology, 18(11), e3000979. 10.1371/journal.pbio.3000979, 33253185 PMC7728398

[bib74] Paquola, C., Vos De Wael, R., Wagstyl, K., Bethlehem, R. A. I., Hong, S.-J., Seidlitz, J., … Bernhardt, B. C. (2019). Microstructural and functional gradients are increasingly dissociated in transmodal cortices. PLOS Biology, 17(5), e3000284. 10.1371/journal.pbio.3000284, 31107870 PMC6544318

[bib75] Park, S., Haak, K. V., Oldham, S., Cho, H., Byeon, K., Park, B., … Hong, S.-J. (2024). A shifting role of thalamocortical connectivity in the emergence of cortical functional organization. Nature Neuroscience, 27(8), 1609–1619. 10.1038/s41593-024-01679-3, 38858608

[bib76] Raichle, M. E., & Mintun, M. A. (2006). Brain work and brain imaging. Annual Review of Neuroscience, 29, 449–476. 10.1146/annurev.neuro.29.051605.112819, 16776593

[bib77] Raut, R. V., Snyder, A. Z., & Raichle, M. E. (2020). Hierarchical dynamics as a macroscopic organizing principle of the human brain. Proceedings of the National Academy of Sciences, 117(34), 20890–20897. 10.1073/pnas.2003383117, 32817467 PMC7456098

[bib78] Rivera-Alba, M., Peng, H., de Polavieja, G. G., & Chklovskii, D. B. (2014). Wiring economy can account for cell body placement across species and brain areas. Current Biology, 24(3), R109–R110. 10.1016/j.cub.2013.12.012, 24502781

[bib79] Rivera-Alba, M., Vitaladevuni, S. N., Mischenko, Y., Lu, Z., Takemura, S.-Y., Scheffer, L., … de Polavieja, G. G. (2011). Wiring economy and volume exclusion determine neuronal placement in the Drosophila brain. Current Biology, 21(23), 2000–2005. 10.1016/j.cub.2011.10.022, 22119527 PMC3244492

[bib80] Roberts, J. A., Perry, A., Lord, A. R., Roberts, G., Mitchell, P. B., Smith, R. E., … Breakspear, M. (2016). The contribution of geometry to the human connectome. NeuroImage, 124, 379–393. 10.1016/j.neuroimage.2015.09.009, 26364864

[bib81] Roberts, J. A., Perry, A., Roberts, G., Mitchell, P. B., & Breakspear, M. (2017). Consistency-based thresholding of the human connectome. NeuroImage, 145, 118–129. 10.1016/j.neuroimage.2016.09.053, 27666386

[bib82] Rubinov, M., Ypma, R. J. F., Watson, C., & Bullmore, E. T. (2015). Wiring cost and topological participation of the mouse brain connectome. Proceedings of the National Academy of Sciences, 112(32), 10032–10037. 10.1073/pnas.1420315112, 26216962 PMC4538676

[bib83] Sato, T. K. (2021). Long-range connections enrich cortical computations. Neuroscience Research, 162, 1–12. 10.1016/j.neures.2020.05.004, 32470355

[bib84] Schaefer, A., Kong, R., Gordon, E. M., Laumann, T. O., Zuo, X.-N., Holmes, A. J., … Yeo, B. T. T. (2018). Local-global parcellation of the human cerebral cortex from intrinsic functional connectivity MRI. Cerebral Cortex, 28(9), 3095–3114. 10.1093/cercor/bhx179, 28981612 PMC6095216

[bib85] Scholtens, L. H., Schmidt, R., de Reus, M. A., & van den Heuvel, M. P. (2014). Linking macroscale graph analytical organization to microscale neuroarchitectonics in the macaque connectome. Journal of Neuroscience, 34(36), 12192–12205. 10.1523/JNEUROSCI.0752-14.2014, 25186762 PMC6608464

[bib86] Schröter, M., Paulsen, O., & Bullmore, E. T. (2017). Micro-connectomics: Probing the organization of neuronal networks at the cellular scale. Nature Reviews Neuroscience, 18(3), 131–146. 10.1038/nrn.2016.182, 28148956

[bib87] Shinn, M., Hu, A., Turner, L., Noble, S., Preller, K. H., Ji, J. L., … Murray, J. D. (2023). Functional brain networks reflect spatial and temporal autocorrelation. Nature Neuroscience, 26(5), 867–878. 10.1038/s41593-023-01299-3, 37095399

[bib88] Smith, R. E., Tournier, J.-D., Calamante, F., & Connelly, A. (2015). SIFT2: Enabling dense quantitative assessment of brain white matter connectivity using streamlines tractography. NeuroImage, 119, 338–351. 10.1016/j.neuroimage.2015.06.092, 26163802

[bib89] Song, H. F., Kennedy, H., & Wang, X.-J. (2014). Spatial embedding of structural similarity in the cerebral cortex. Proceedings of the National Academy of Sciences, 111(46), 16580–16585. 10.1073/pnas.1414153111, 25368200 PMC4246295

[bib90] Sotiropoulos, S. N., & Zalesky, A. (2019). Building connectomes using diffusion MRI: Why, how and but. NMR in Biomedicine, 32(4), e3752. 10.1002/nbm.3752, 28654718 PMC6491971

[bib91] Sporns, O., & Betzel, R. F. (2016). Modular brain networks. Annual Review of Psychology, 67, 613–640. 10.1146/annurev-psych-122414-033634, 26393868 PMC4782188

[bib92] Sydnor, V. J., Larsen, B., Bassett, D. S., Alexander-Bloch, A., Fair, D. A., Liston, C., … Satterthwaite, T. D. (2021). Neurodevelopment of the association cortices: Patterns, mechanisms, and implications for psychopathology. Neuron, 109(18), 2820–2846. 10.1016/j.neuron.2021.06.016, 34270921 PMC8448958

[bib93] Tian, Y., Margulies, D. S., Breakspear, M., & Zalesky, A. (2020). Topographic organization of the human subcortex unveiled with functional connectivity gradients. Nature Neuroscience, 23(11), 1421–1432. 10.1038/s41593-020-00711-6, 32989295

[bib94] van den Heuvel, M. P., Bullmore, E. T., & Sporns, O. (2016). Comparative connectomics. Trends in Cognitive Sciences, 20(5), 345–361. 10.1016/j.tics.2016.03.001, 27026480

[bib95] van den Heuvel, M. P., Kahn, R. S., Goñi, J., & Sporns, O. (2012). High-cost, high-capacity backbone for global brain communication. Proceedings of the National Academy of Sciences, 109(28), 11372–11377. 10.1073/pnas.1203593109, 22711833 PMC3396547

[bib96] van den Heuvel, M. P., Scholtens, L. H., Feldman Barrett, L., Hilgetag, C. C., & de Reus, M. A. (2015). Bridging cytoarchitectonics and connectomics in human cerebral cortex. Journal of Neuroscience, 35(41), 13943–13948. 10.1523/JNEUROSCI.2630-15.2015, 26468195 PMC6608182

[bib97] van den Heuvel, M. P., & Sporns, O. (2011). Rich-club organization of the human connectome. Journal of Neuroscience, 31(44), 15775–15786. 10.1523/JNEUROSCI.3539-11.2011, 22049421 PMC6623027

[bib98] van den Heuvel, M. P., & Sporns, O. (2013). Network hubs in the human brain. Trends in Cognitive Sciences, 17(12), 683–696. 10.1016/j.tics.2013.09.012, 24231140

[bib99] Vértes, P. E. (2023). Computational models of typical and atypical brain network development. Biological Psychiatry, 93(5), 464–470. 10.1016/j.biopsych.2022.11.012, 36593135

[bib100] Vértes, P. E., Alexander-Bloch, A. F., Gogtay, N., Giedd, J. N., Rapoport, J. L., & Bullmore, E. T. (2012). Simple models of human brain functional networks. Proceedings of the National Academy of Sciences, 109(15), 5868–5873. 10.1073/pnas.1111738109, 22467830 PMC3326510

[bib101] Vohryzek, J., Sanz-Perl, Y., Kringelbach, M. L., & Deco, G. (2025). Human brain dynamics are shaped by rare long-range connections over and above cortical geometry. Proceedings of the National Academy of Sciences, 122(1), e2415102122. 10.1073/pnas.2415102122, 39752525 PMC11725837

[bib102] Watts, D. J., & Strogatz, S. H. (1998). Collective dynamics of ‘small-world’ networks. Nature, 393(6684), 440–442. 10.1038/30918, 9623998

[bib103] Wei, Y., Scholtens, L. H., Turk, E., & van den Heuvel, M. P. (2018). Multiscale examination of cytoarchitectonic similarity and human brain connectivity. Network Neuroscience, 3(1), 124–137. 10.1162/netn_a_00057, 30793077 PMC6372019

[bib104] Zhang, X., Braun, U., Harneit, A., Zang, Z., Geiger, L. S., Betzel, R. F., … Tost, H. (2021). Generative network models of altered structural brain connectivity in schizophrenia. NeuroImage, 225, 117510. 10.1016/j.neuroimage.2020.117510, 33160087

