# Supplementary material for: Coming up short: Generative network models fail to accurately capture long-range connectivity
Source: Netw Neurosci. 2025 Nov 20;9(4):1377–400. doi: 10.1162/NETN.a.35 (PMC12635836; doi:10.1162/NETN.a.35)
Supplement: Supplementary file 1 [file netn-9-4-1377-s001.pdf]

Supplementary material for “Coming up short: generative network models fail to accurately capture long-range connectivity”

Stuart Oldham[1,2], Alex Fornito[2] & Gareth Ball[1,3]

1. Developmental Imaging, Murdoch Children’s Research Institute, The Royal Children’s Hospital Melbourne
2. The Turner Institute for Brain and Mental Health, School of Psychological Sciences and Monash Biomedical Imaging, Monash University, Clayton, Australia
3. Department of Paediatrics, University of Melbourne, Melbourne

Corresponding author
Dr Stuart Oldham
Developmental Imaging,
Murdoch Children’s Research Institute
Parkville 3052 VIC
Australia
stuart.oldham@mcri.edu.au

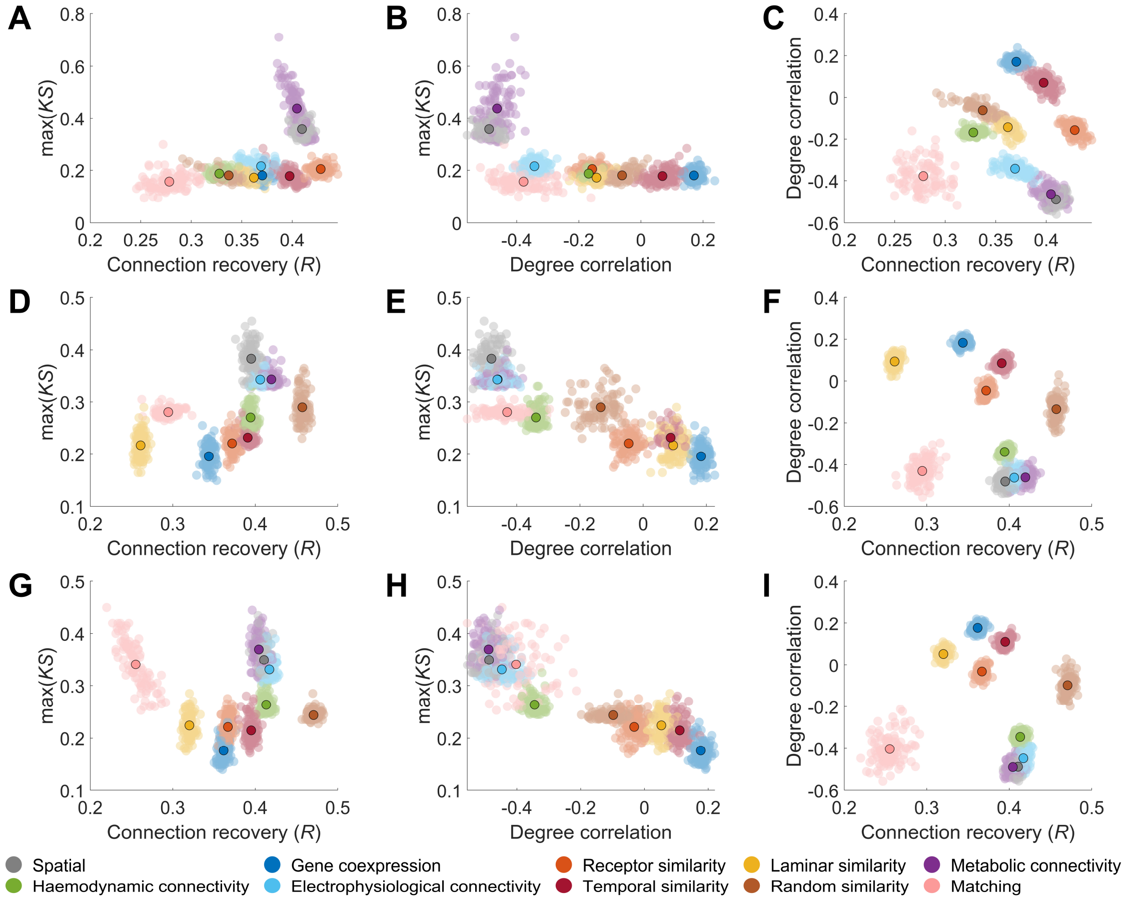

Figure S1. Performance of different formulations of generative network models in $\max(\mathrm{KS})$, connections recovered, and similarity of the degree distribution. Generative network models formulated using an additive form and power-law distance decay showing the: **(A)** relationship between $\max(KS)$ and connections recovered; **(B)** relationship between $\max(KS)$ and the correlation between empirical and model degree; and **(C)** relationship between $\max(KS)$ and the correlation between empirical and model degree. Generative network models formulated using a multiplicative form and exponential distance decay showing the: **(D)** relationship between $\max(KS)$ and connections recovered; **(E)** relationship between $\max(KS)$ and the correlation between empirical and model degree; and **(F)** relationship between $\max(KS)$ and the correlation between empirical and model degree. Generative network models formulated using a multiplicative form and power-law distance decay showing the: **(G)** relationship between $\max(KS)$ and connections recovered; **(H)** relationship between $\max(KS)$ and the correlation between empirical and model degree; and **(I)** relationship between $\max(KS)$ and the correlation between empirical and model degree.

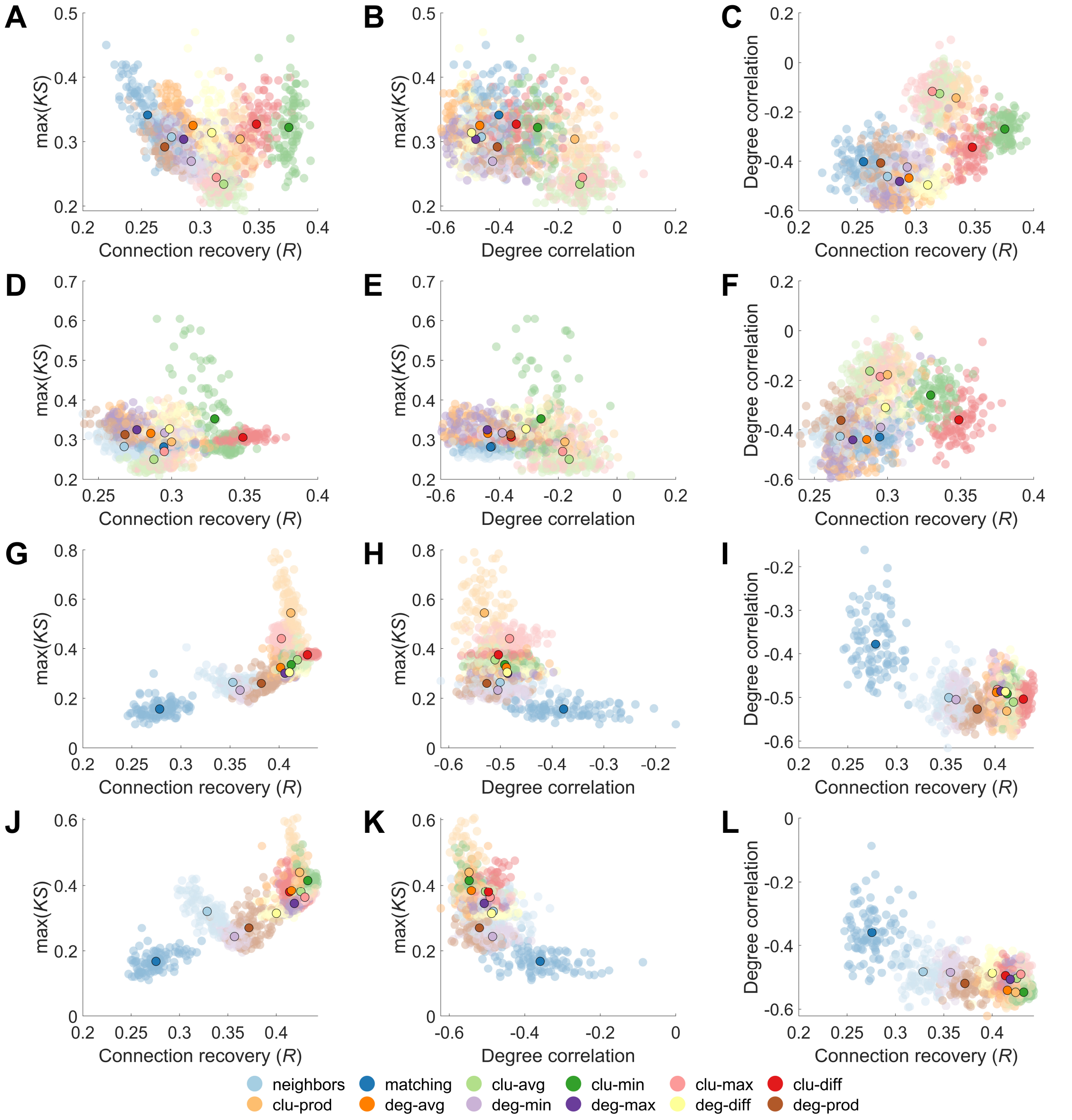

Figure S2. Performance of different topological generative network models in $\max(KS)$, connections recovered, and similarity of the degree distribution. Generative network models formulated using a multiplicative form and power-law distance decay showing the: **(A)** relationship between $\max(KS)$ and connections recovered; **(B)** relationship between $\max(KS)$ and the correlation between empirical and model degree; and **(C)** relationship between $\max(KS)$ and the correlation between empirical and model degree. Generative network models formulated using a multiplicative form and exponential distance decay showing the: **(D)** Relationship between $\max(KS)$ and connections recovered; **(E)** relationship between $\max(KS)$ and the correlation between empirical and model degree; and **(F)** relationship between $\max(KS)$ and the correlation between empirical and model degree. Generative network models formulated using an additive form and power-law distance decay showing the: **(G)** relationship between $\max(KS)$ and connections recovered; **(H)** relationship between $\max(KS)$ and the correlation between empirical and model degree; and **(I)** relationship between $\max(KS)$ and the correlation between empirical and model degree. Generative network models formulated using an additive form and exponential distance decay showing the: **(J)** Relationship between $\max(KS)$ and connections recovered; **(K)** relationship between $\max(KS)$ and the correlation between empirical and model degree; and **(L)** relationship between $\max(KS)$ and the correlation between empirical and model degree.

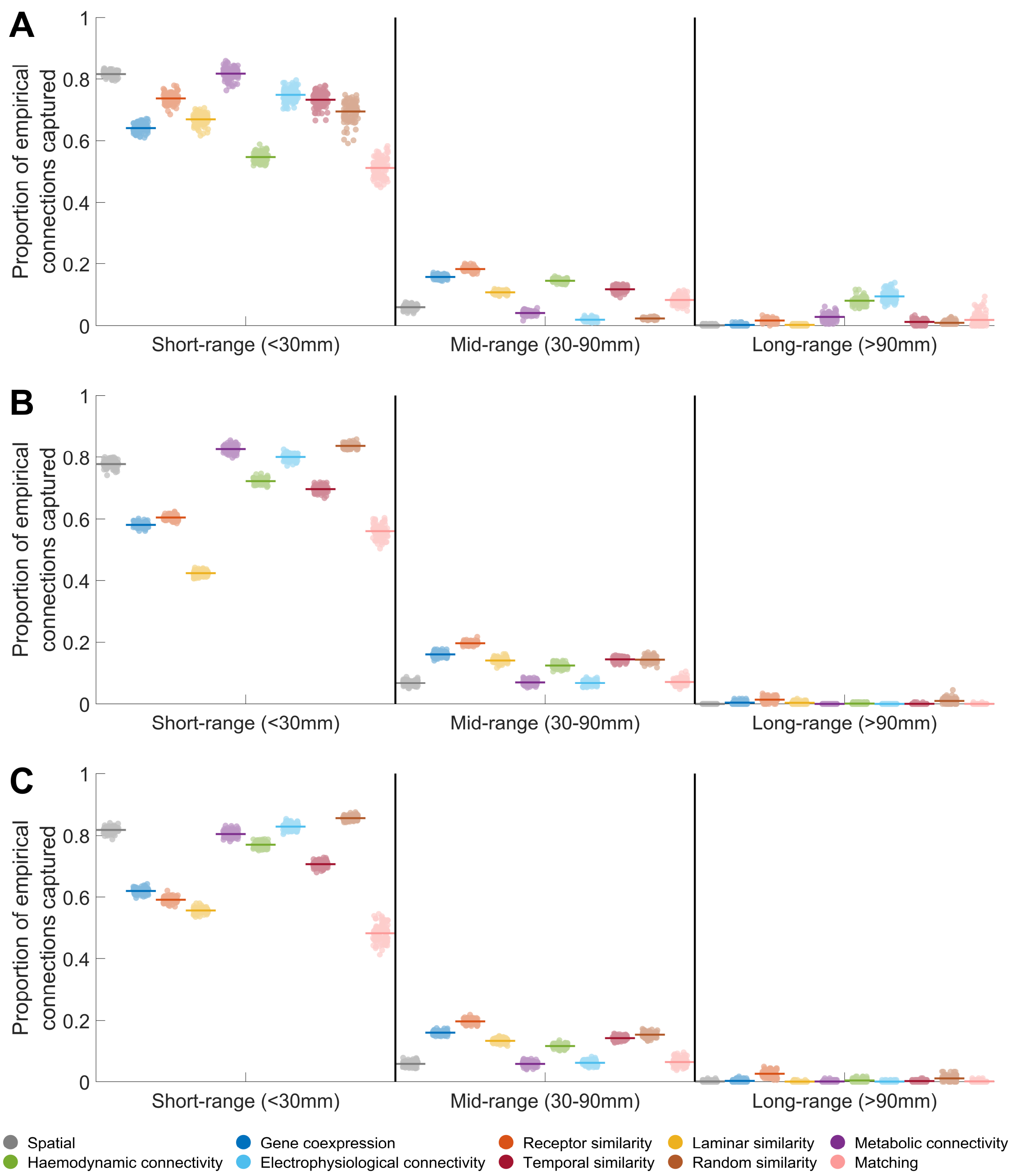

Figure S3. Connections captured at different distance thresholds for different generative network model formulations. Proportion of empirical short-range, mid-range, and long-range connections captured by the best fitting (lowest $\max(KS)$) generative network models for the: **(A)** additive and power-law distance decay formulation; **(B)** multiplicative and exponential distance decay formulation; **(C)** multiplicative and power-law distance decay formulation. The coloured line indicates the average overlap, while each point indicates the result for an individual model network.

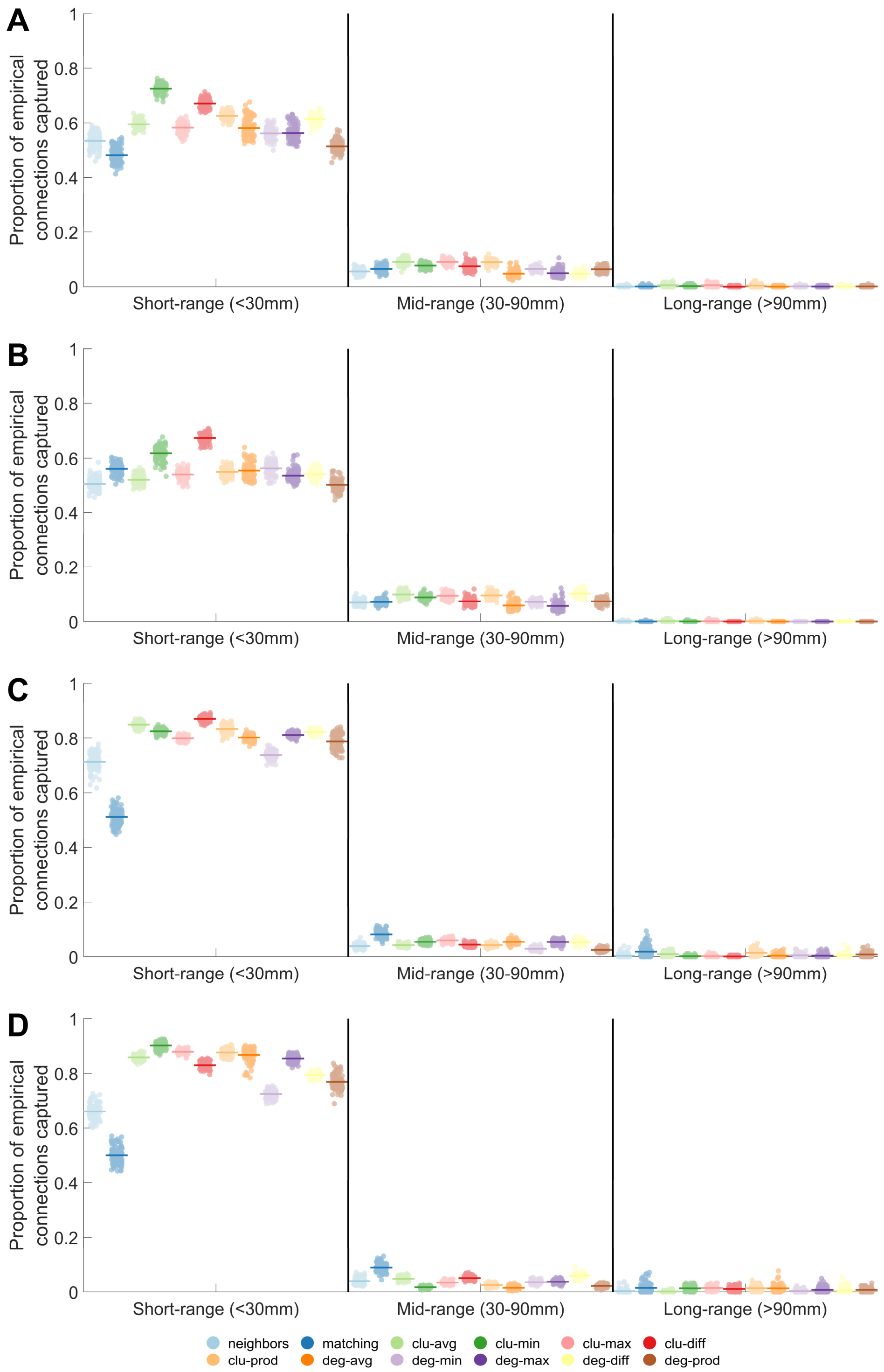

Figure S4. Connections captured at different distance thresholds for topological generative network models. Proportion of empirical short-range, mid-range, and long-range connections captured by the best fitting (lowest $\max(KS)$) topological generative network models for the: **(A)** multiplicative and power-law distance decay formulation; **(B)** multiplicative and exponential distance decay formulation; **(C)** additive and power-law distance decay formulation; **(D)** additive and exponential distance decay formulation. The coloured line indicates the average overlap, while each point indicates the result for an individual model network.

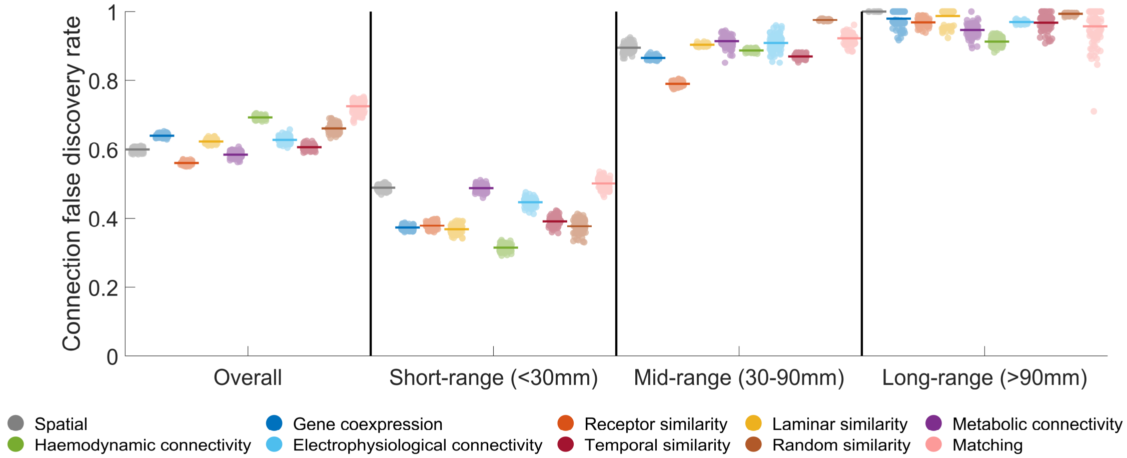

Figure S5. False discovery rate for connections at different distance thresholds. The false discovery rate (i.e., the proportion of connections generated by the model which were not found in the empirical data) is computed for all, short-range, mid-range, and long-range connections for the 10 main GNMs that used the additive, exponential decay formulation.

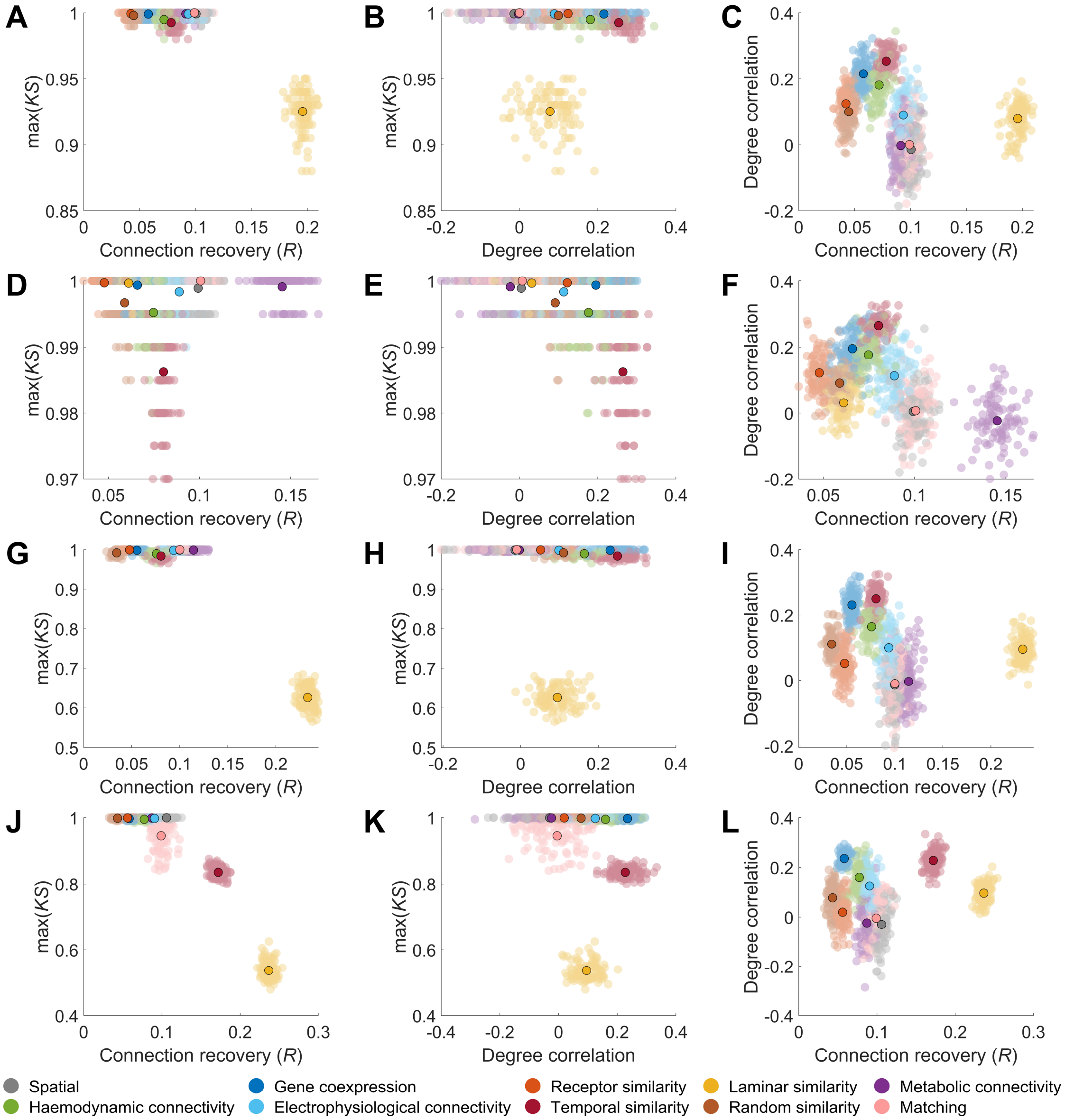

Figure S6. Analysis of generative network models with the strongest degree correlations, evaluated through $\max(KS)$, connections recovered, and degree correlation. (**A-C**) Models using an additive form with exponential distance decay: (**A**) Relationship between $\max(KS)$ and connections recovered. (**B**) Relationship between $\max(KS)$ and the correlation between empirical and model degree. (**C**) Relationship between $max(KS)$ and the correlation between empirical and model degree. (**D-F**) Models using a power-law distance decay: (**D**) Relationship between $\max(KS)$ and connections recovered. (**E**) Relationship between $\max(KS)$ and the correlation between empirical and model degree. (**F**) Relationship between $\max(KS)$ and the correlation between empirical and model degree. (**G-I**) Models using a multiplicative form with exponential distance decay: (**G**) Relationship between $\max(KS)$ and connections recovered. (**H**) Relationship between $\max(KS)$ and the correlation between empirical and model degree. (**I**) Relationship between $\max(KS)$ and the correlation between empirical and model degree. (**J-L**) Models using a multiplicative form with power-law distance decay: (**J**) Relationship between $\max(KS)$ and connections recovered. (**K**) Relationship between $\max(KS)$ and the correlation between empirical and model degree. (**L**) Relationship between $\max(KS)$ and the correlation between empirical and model degree.

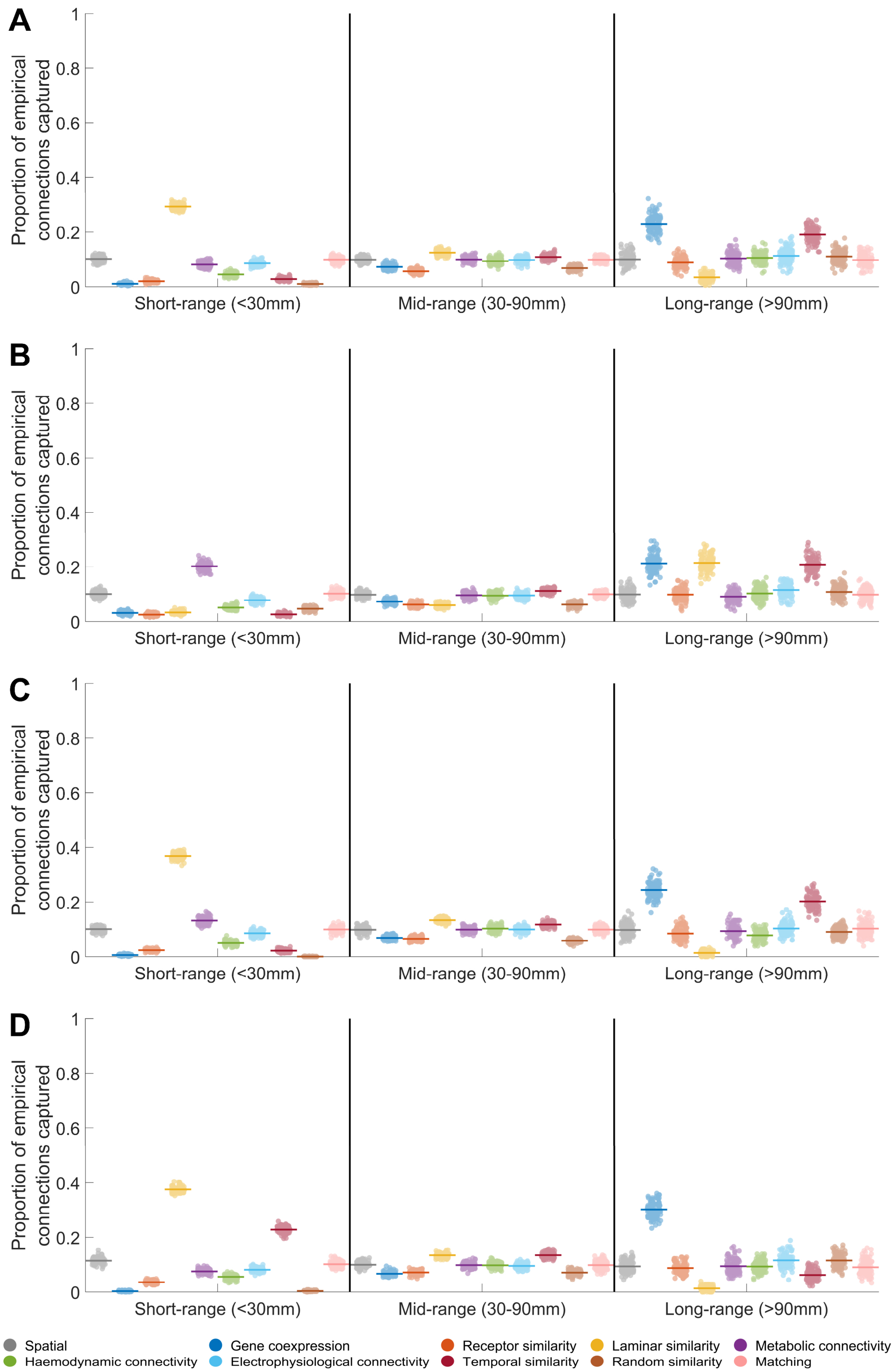

Figure S7. Connections captured at different distance thresholds for generative network models with the strongest degree correlations. Proportion of empirical short-range, mid-range, and long-range connections captured by the generative network models with the strongest degree correlations for the: **(A)** additive and exponential distance decay formulation; **(B)** additive and power-law distance decay formulation; **(C)** multiplicative and exponential distance decay formulation; **(D)** multiplicative and power-law distance decay formulation. The coloured line indicates the average overlap, while each point indicates the result for an individual model network.

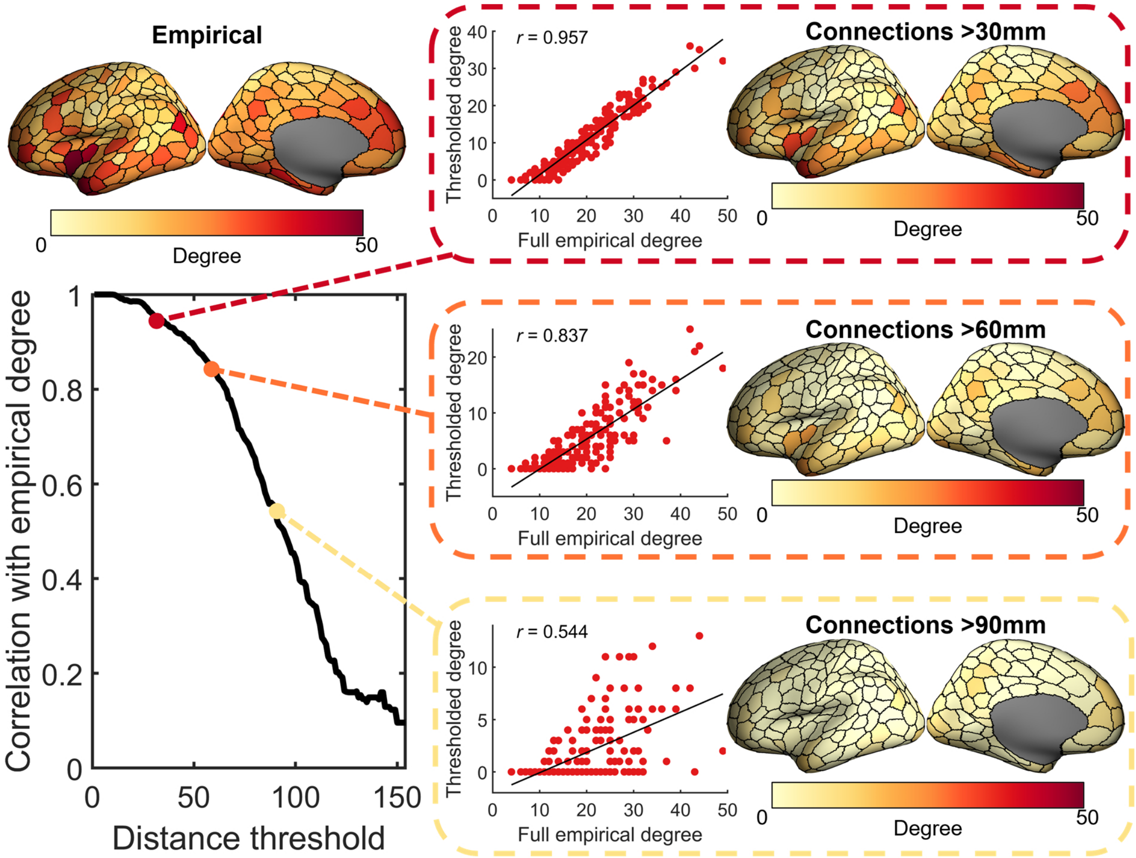

Figure S8. Similarity of the empirical degree sequence at different distance thresholds. Degree is calculated only using connections with a length greater than the current distance threshold. The thresholded degree is then correlated with the full empirical degree (i.e., degree calculated when using all empirical connections).

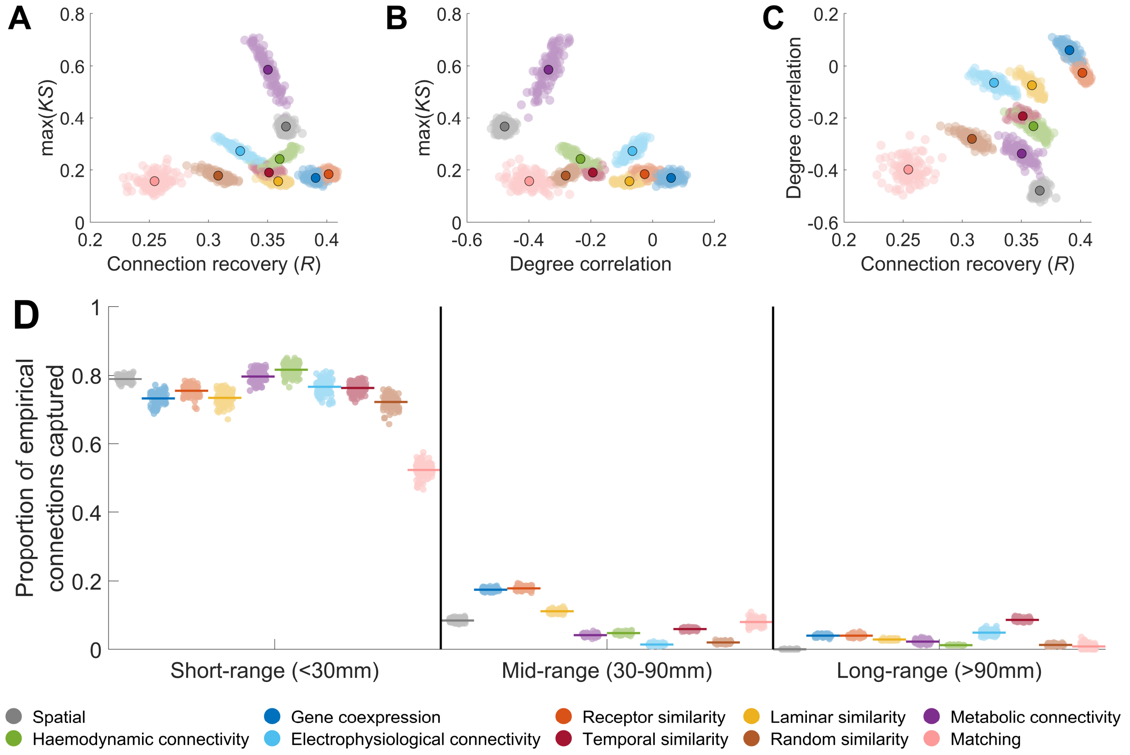

Figure S9. Performance of generative network models in capturing topological and topographical properties in the whole-brain group consensus network. (A) Relationship between $\max(KS)$ and connection recovery of synthetic model networks. **(B)** Relationship between $\max(KS)$ and the correlation between empirical and model node degree. **(C)** Relationship between connection recovery and the correlation between empirical and model degree. In each plot, the outlined point indicates the average across model runs for different model formulations. (**D**) Proportion of empirical short-range, mid-range, and long-range connections captured by the best fitting generative network models. The coloured line indicates the average overlap, while each point indicates the result for an individual model network.

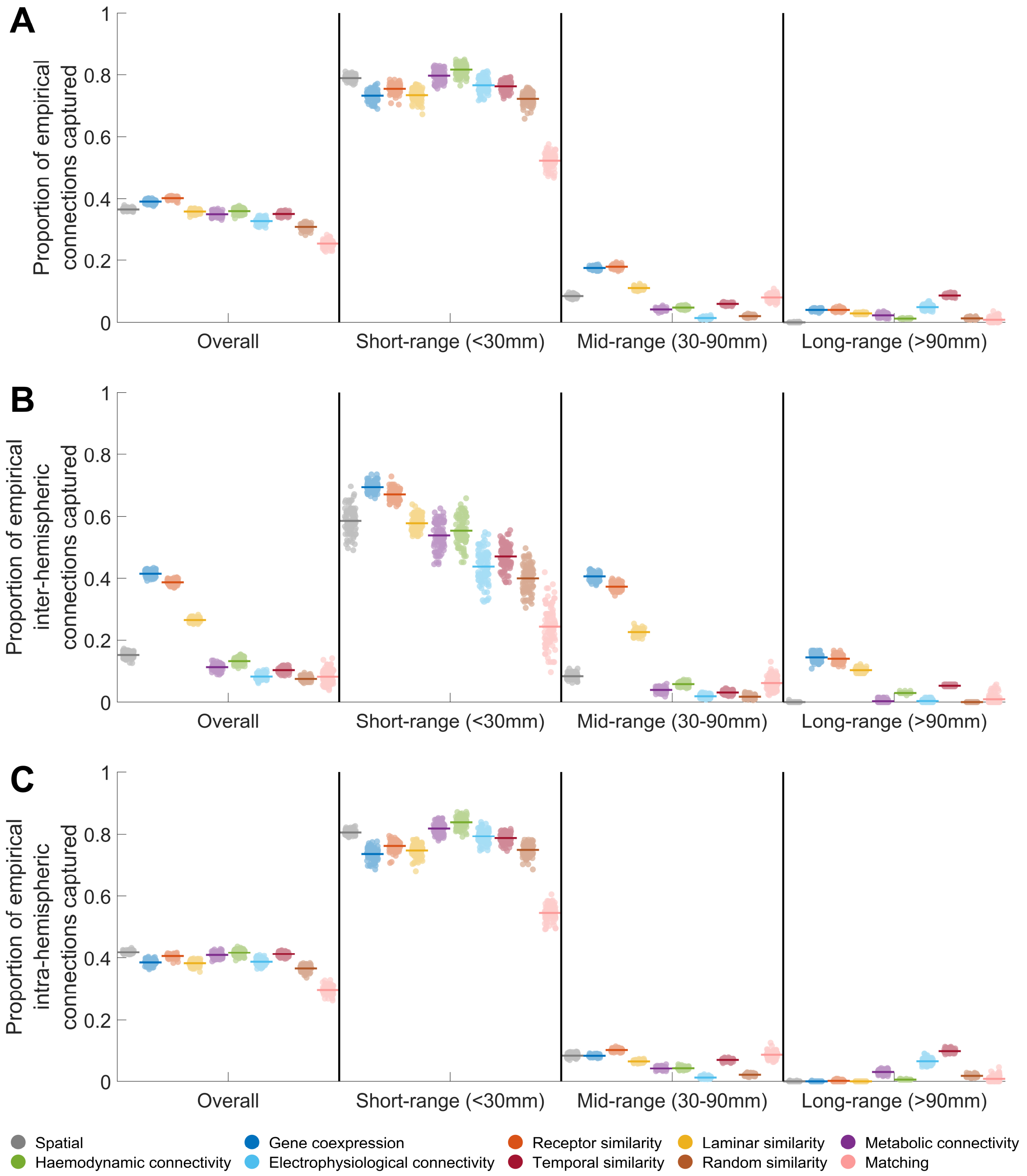

Figure S10. Connection recovery for all, inter-hemispheric, and intra-hemispheric connections for the whole-brain group consensus network by the additive, exponential decay GNMs. Proportion of empirical connections captured for (**A**) all connection types; (**B**) inter-hemispherical connections; (**C**) intra-hemispheric connections across different distance thresholds (all, short-range, mid-range, and long-range). Note that no short-range inter-hemispheric connections exist, and the stratification for the mid-range inter-hemispheric connections is because few such connections exist empirically.

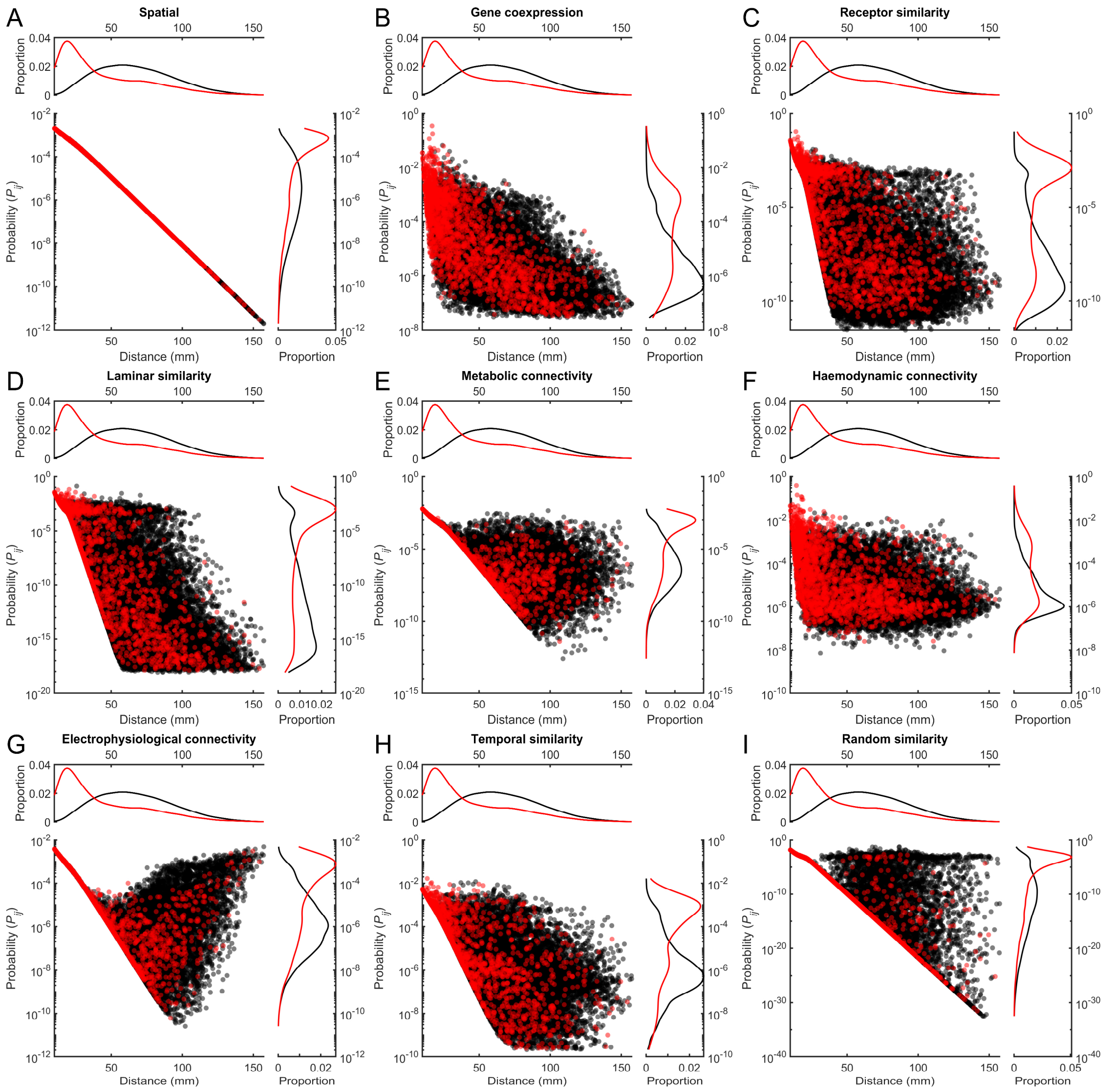

Figure S11. Connection probabilities in generative network models. Shown are connection probabilities for the network with the lowest $\max(KS)$ for all GNMs besides the *Matching* model (see Figure 4 in main text). The connection probabilities are the mean probability assigned to each connection across all timepoints of the model. Red points indicate structural connections (i.e., connections that exist in the empirical data) while black points indicate non-structurally connected regions. Kernal density plots for the structural and non-structural connections are shown for the distributions of distance/length and probability against the respective axis.

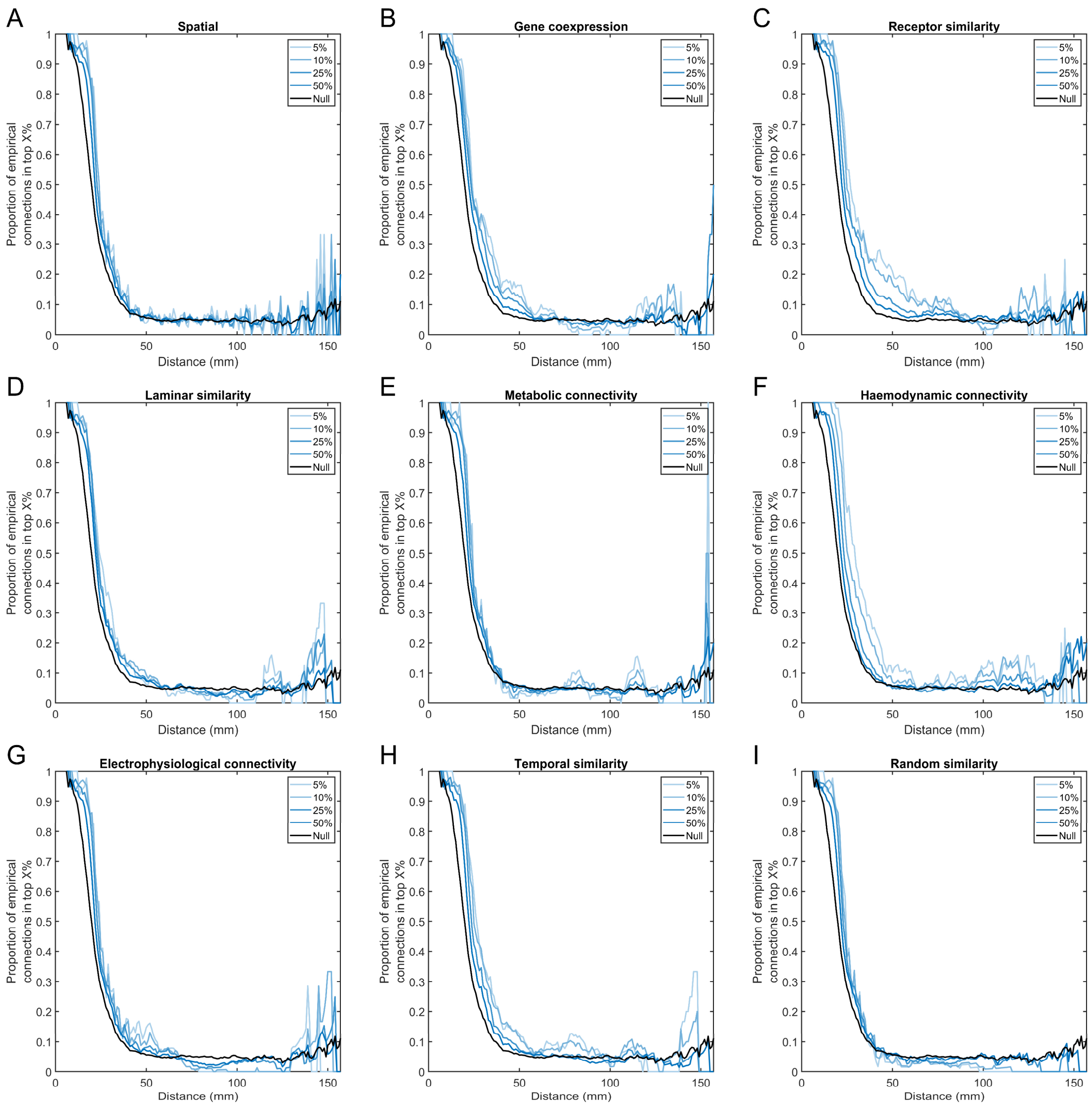

Figure S12. Most probable connections at different distances across generative network models. Using the sliding window analysis, at each distance the top X% of connections with the highest probability are found. The proportion of these which are empirical connections is then calculated. The "null" line indicates the proportion of empirical structural connections that exist at that distance (i.e., the probability of selecting an empirical structural connection if all connections at a given distance were equally probable).

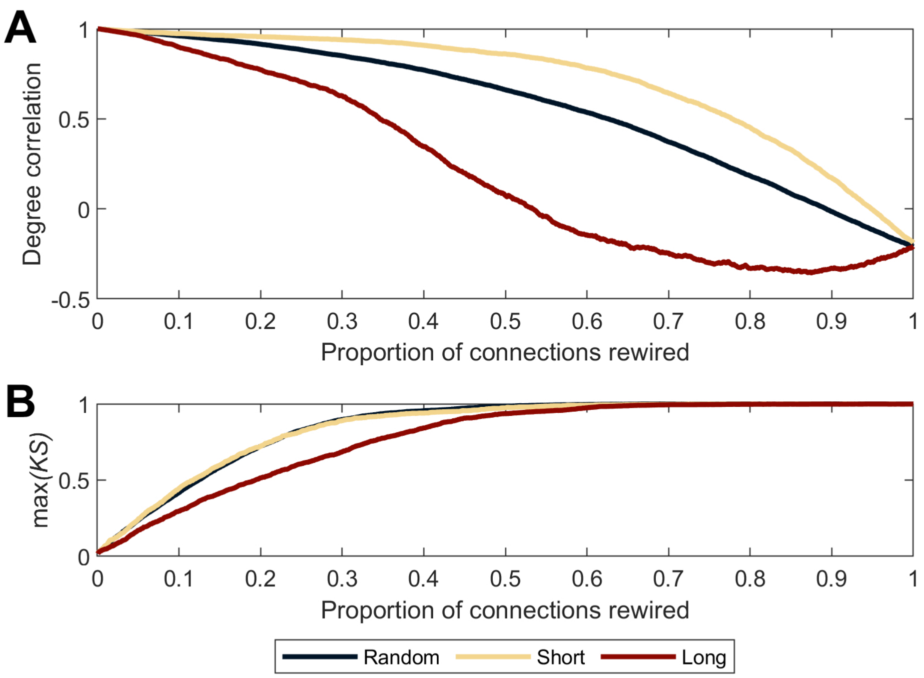

Figure S13. Effect of iteratively rewiring connections on the degree correlation. (A) The proportion of connections rewired and its effect on the degree correlation (i.e., correlation between node degree in the original and rewired network) for different rewiring algorithms. **(B)** The proportion of connections rewired and its effect on $\max(KS)$ for different rewiring algorithms. The *random* algorithm rewired connections at random but of a similar length; *short* rewired connections from shortest to longest connections with those of a similar length and; *long* rewired connections from longest to shortest connections with those of a similar length.

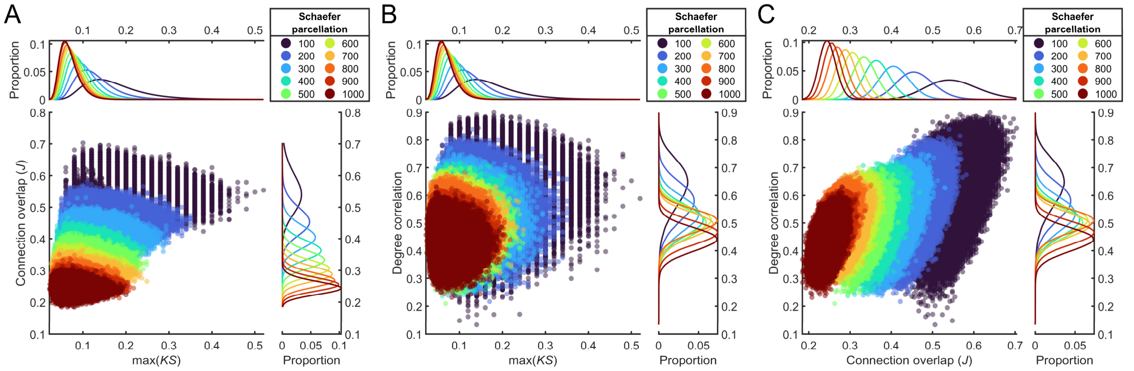

Figure S14. Performance of $\max(KS)$, connection overlap, and similarity of the degree distribution when comparing empirical networks produced using deterministic tractography. (A) Relationship between $max(KS)$ and connection overlap. **(B)** Relationship between $max(KS)$ and the correlation between two empirical networks nodal degree. **(C)** Relationship between connection overlap and the correlation between two empirical networks nodal degree. The kernel density plots show the distributions for each parcellation on the respective feature.

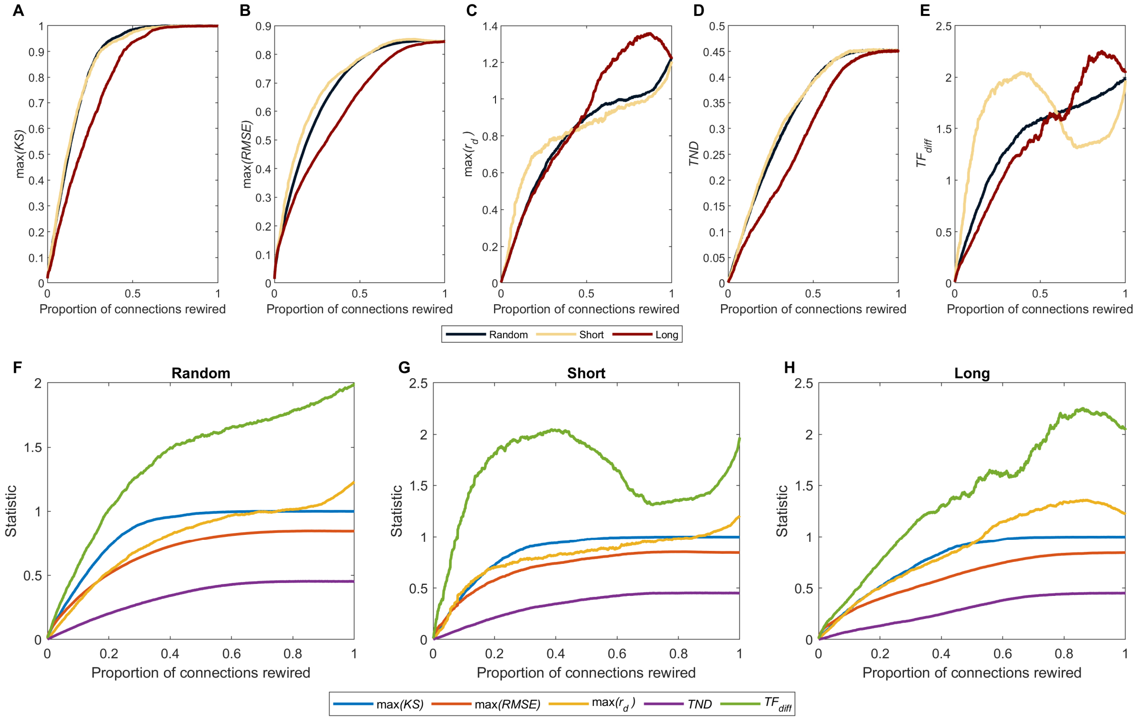

Figure S15. Effect of iteratively rewiring edges on different network similarity measures. The proportion of connections rewired and its effect on **(A)** $\max(KS)$; **(B)** $\max(RMSE)$; **(C)** $max(r_d)$; **(D)** TND; **(E)** and TF_{diff} for different rewiring algorithms. Each statistic has been normalised to its value obtained at 100% rewiring. Comparison of the different similarity measures for the: **(F)** *random*: **(G)** *short*; **(H)** *long*. The random algorithm rewired connections at random but of a similar length; *short* rewired connections from shortest to longest connections with those of a similar length; *long* rewired connections from longest to shortest connections with those of a similar length.

Table S1

Definitions of topological coupling terms

Name	F_{ij}	Description
clu-avg	$\left(\frac{c_i}{2} + \frac{c_j}{2}\right)$	Mean clustering coefficient of nodes i and j
clu-diff	$\lvert c_i - c_j \rvert$	Absolute difference between the clustering coefficients of nodes i and j
clu-max	$\max[c_i, c_j]$	Maximum clustering coefficient of nodes i and j
clu-min	$\min[c_i, c_j]$	Minimum clustering coefficient of nodes i and j
clu-prod	$c_i c_j$	Product of the clustering coefficients of nodes i and j
deg-avg	$\left(\frac{k_i}{2} + \frac{k_j}{2}\right)$	Mean degree of nodes i and j
deg-diff	$\lvert k_i - k_j \rvert$	Absolute difference between the degree of nodes i and j
deg-max	$\max[k_i, k_j]$	Maximum degree of nodes i and j
deg-min	$\min[k_i, k_j]$	Minimum degree of nodes i and j
deg-prod	$k_i k_j$	Product of the degrees of nodes i and j
matching	$\frac{2 \sum_k A_{ik} A_{jk}}{k_i + k_j - 2A_{ij}}$	The proportion of neighbors shared by nodes i and j
neighbors	$\sum_k A_{ik} A_{jk}$	The number of nodes neighboring both i and j

k_i = degree of node i, c_i = clustering coefficient of node i, A = adjacency matrix. Note that when topology was used in the model, to prevent undefined values from occurring (such as $F_{ij} = 0$) we add $\varepsilon = 10^{-6}$ to F_{ij}

Table S2

Different measures of network topological and topographical similarity

Network similarity measure	Formula	Definition
$\max(KS)$	$\max(\{KS_k, KS_c, KS_b, KS_e\})$	The maximum Kolmogorov-Smirnov (KS) statistic among: nodal degree k, clustering c, betweenness b, and edge connection length e
TND	$\sqrt{(\epsilon - \hat{\epsilon})^2 + (\Omega - \hat{\Omega})^2 + (Q - \hat{Q})^2 + (T - \hat{T})^2}$	Euclidean distance between four global topological features: efficiency ϵ, diffusion efficiency Ω, modularity Q, transitivity T
TF_{diff}	$\sqrt{\sum_i \sum_j \left(TF(A)_{ij} - TF(\hat{A})_{ij}\right)^2}$	Frobenius/Euclidean norm of the difference between the topological correlation matrices TF of two networks (Akarca et al., 2022)
$\max(RMSE)$	$\max(\{RMSE_k, RMSE_c, RMSE_h, RMSE_d\})$	The maximum root-mean-square-error (RMSE) of degree, clustering, harmonic centrality, and nodal distance
$\max(r_d)$	$\max(\{r_d(k), r_d(c), r_d(b), r_d(d)\})$	The maximum Pearson's correlation distance r_d among: nodal degree k, clustering c, betweenness b, and nodal distance d
Connection recovery (R)	$\frac{\lvert A \cap \hat{A} \rvert}{\lvert A \rvert}$	Proportion of connections in $\hat{A}$ that overlap with those in A
Connection overlap (J)	$\frac{\lvert A \cap \hat{A} \rvert}{\lvert A \cup \hat{A} \rvert}$	Proportion of overlapping connections between A and $\hat{A}$ over the total number of unique connections present in either network

Note. The ^ symbol indicates either a secondary adjacency matrix, or a feature derived from that matrix. $TF(A)$ is a 6×6 matrix of the Pearson correlations between the nodal distributions of degree, clustering, betweenness, nodal distance, mean matching, and closeness for the adjacency matrix A. The RMSE of harmonic centrality was used instead of the RMSE for betweenness ($RMSE_b$) as $RMSE_b$ was consistently much higher than the other RMSE values and would cause the measure to become entirely driven by differences in nodal betweenness values.

Table S3

Network measures/miscellaneous equations

Measure	Formula	Definition
KS	$KS_F = \max_x(\lvert G(x) - \hat{G}(x)\rvert)$	The maximum discrepancy between the cumulative empirical distribution functions G and $\hat{G}$ (Dudley, 2015)
RMSE	$RMSE_x = \frac{\sqrt{\frac{1}{n}\sum_i^n (x_i - \hat{x}_i)^2}}{\langle x \rangle}$	The root-mean-square-error (RMSE) of feature x, normalised by the mean of x
Degree	$k_i = \sum_{j \neq i} A_{ij}$	Number of connections a node has
Clustering	$c_i = \frac{\sum_{j,k} A_{ij} A_{jk} A_{ki}}{k_i(k_i - 1)}$	Average number of pairs of neighbours of a node that are connected (Watts & Strogatz, 1998)
Betweenness	$b_i = \sum_{u \neq i, u \neq v, v \neq i} \frac{\rho_{uv}(i)}{\rho_{uv}}$	The number of shortest-paths a node takes part in (Freeman, 1977)
Nodal distance	$d_i = \frac{1}{k_i} \sum_j D_{ij} A_{ij}$	The mean length of a node's connections
Harmonic (closeness) centrality	$h_i = \frac{1}{N-1} \sum_i \frac{1}{L_{ij}}$	The average of inverse shortest path lengths (Latora & Marchiori, 2001; Marchiori & Latora, 2000)
Matching index	$M_{ij} = \frac{\sum_k A_{ik} A_{jk}}{k_i + k_j - 2A_{ij}}$	The proportion of identical connections of two nodes normalised by the total number of connections belonging to the two nodes
Shortest-path efficiency	$\epsilon = \frac{1}{N(N-1)} \sum_{i \neq j} \frac{1}{L_{ij}}$	The average length of the paths between all nodes (Latora & Marchiori, 2001)
Diffusion efficiency	$\Omega = \frac{1}{N(N-1)} \sum_{i \neq j} \frac{1}{H_{ij}}$	The average length of random-walks between all nodes (Goñi et al., 2013)
Modularity	$Q = \frac{1}{2E} \sum_{i,j} \left(A_{ij} - \frac{k_i k_j}{2E}\right) \delta(m_i, m_j)$	The proportion of connections that occur within modules relative to that expect if connections were placed randomly (Newman & Girvan, 2004)
Transitivity	$T = \frac{\sum_{i,j,k} A_{ij} A_{jk} A_{ki}}{\sum_i k_i(k_i - 1)}$	The number of closed triplets divided by the number of all possible triplets (Newman, 2003; Wasserman & Faust, 1994)

Note. $G(x)$ and $\hat{G}(x)$ are the proportion of values of the respective features G and $\hat{G}$ that are less than or equal to x; ρ_{uv} is the number of shortest/ geodesic paths between nodes u and v; $\rho_{uv}(i)$ is the number of shortest paths between nodes u and v which pass through node i; L_{ij} is the length of the shortest path between node i and j; H_{ij} is the mean-first passage time from node i to node j; E is the number of edges in the network; $\delta(m_i, m_j)$ is the Kronecker delta function which is equal to one if nodes i and j are part of the same module and zero otherwise. When calculating the RMSE for two empirical networks, given both x and $\hat{x}$ correspond to observed values, instead of dividing by $\langle x \rangle$, the numerator was divided by $\frac{\langle x \rangle + \langle \hat{x} \rangle}{2}$ as to consider the averages of both sets of empirical observations. The mean matching index of node i can be calculated as $\frac{1}{N}\sum_j M_{ij}$ to obtain a nodal measure of the index. For identifying modules, modularity maximisation was used. Diffusion efficiency was only calculated in the largest component to prevent

Akarca, D., Dunn, A. W. E., Hornauer, P. J., Ronchi, S., Fiscella, M., Wang, C., Terrigno, M., Jagasia, R., Vértes, P. E., Mierau, S. B., Paulsen, O., Eglen, S. J., Hierlemann, A., Astle, D. E., & Schröter, M. (2022). *Homophilic wiring principles underpin neuronal network topology* in vitro. https://doi.org/10.1101/2022.03.09.483605

Dudley, R. M. (2015). *KOLMOGOROV–SMIRNOV AND MANN–WHITNEY–WILCOXON TESTS*. https://math.mit.edu/~rmd/465/edf-ks.pdf

Freeman, L. C. (1977). A Set of Measures of Centrality Based on Betweenness. *Sociometry*, *40*(1), 35–41.

Goñi, J., Avena-Koenigsberger, A., Velez de Mendizabal, N., van den Heuvel, M. P., Betzel, R. F., & Sporns, O. (2013). Exploring the Morphospace of Communication Efficiency in Complex Networks. *PLoS ONE*, *8*(3), e58070. https://doi.org/10.1371/journal.pone.0058070

Latora, V., & Marchiori, M. (2001). Efficient behavior of small-world networks. *Physical Review Letters*, *87*(19), 198701. https://doi.org/10.1103/PhysRevLett.87.198701

Marchiori, M., & Latora, V. (2000). Harmony in the small-world. *Physica A: Statistical Mechanics and Its Applications*, *285*(3–4), 539–546. https://doi.org/10.1016/S0378-4371(00)00311-3

Newman, M. E. J. (2003). The Structure and Function of Complex Networks. *SIAM Review*, *45*(2), 167–256. https://doi.org/10.1137/S003614450342480

Newman, M. E. J., & Girvan, M. (2004). Finding and evaluating community structure in networks. *Physical Review E*, *69*(2), 026113. https://doi.org/10.1103/PhysRevE.69.026113

Wasserman, S., & Faust, K. (1994). *Social network analysis: Methods and applications*.

Watts, D. J., & Strogatz, S. H. (1998). Collective dynamics of’small-world’ networks. *Nature*, *393*(6684), 440–442. https://doi.org/Doi 10.1038/30918
